# Heat Treatment Effects on Additively Manufactured Polymer Matrix Composites

**DOI:** 10.3390/polym18192446

**Published:** 2026-10-08

**Authors:** Germán Omar Barrionuevo, Felipe Fernández, Yuvaraj Ravishankar Chappidi, Jorge Ramos-Grez, Wilson Navas-Pinto, Jonathan D. Reyes-Ortiz, Duncan E. Cree

**Affiliations:** 1 Department of Mechanical Engineering, Universidad San Francisco de Quito USFQ, Diego de Robles y Vía Interoceánica, Quito P.O. Box 17-0901, Ecuador; 2 Faculty of Materials Science and Technology, Technische Universität Bergakademie Freiberg, Leipziger Straße 34, 09599 Freiberg, Germany; 3 Department of Mechanical and Metallurgical Engineering, School of Engineering, Pontificia Universidad Católica de Chile, Av. Vicuña Mackenna, Santiago 4860, Chile; 4 Departamento de Ciencias de la Energía y Mecánica, Universidad de las Fuerzas Armadas ESPE, Sangolquí 171103, Ecuador; 5 Centro de Nanociencia y Nanotecnología, Universidad de las Fuerzas Armadas ESPE, Sangolquí 171103, Ecuador; 6 Department of Materials Science and Engineering, Carnegie Mellon University, Pittsburgh, PA 15213, USA; 7 Department of Mechanical Engineering, McMaster University, 1280 Main Street West, Hamilton, ON L8S 4L8, Canada

**Keywords:** heat treatment, additive manufacturing, polymer matrix composites, mechanical properties

## Abstract

Additive manufacturing (AM) of polymer matrix composites (PMCs) enables the fabrication of complex, structurally efficient parts, but the layer-wise thermal history intrinsic to processes such as material extrusion, vat photopolymerization, powder bed fusion, and direct ink writing leaves as-printed components in a metastable state: sub-equilibrium crystallinity or incomplete cure, interlayer porosity, weak interlaminar bonding, and locked-in residual stress. Post-fabrication heat treatments have positioned themselves as an attractive and straightforward strategy to address these deficiencies; however, the reported outcomes vary widely across material systems and treatment protocols. This review consolidates and analyzes the existing literature on heat treatment effects in AM-PMCs by organizing the different reported strategies into a taxonomy encompassing free and constrained annealing, pressure-assisted consolidation (including hot isostatic pressing and hot pressing), and post-curing processes for thermoset and photopolymers. The comprehensive literature review allowed identification of four coupled microstructural mechanisms, which govern the resulting property changes after the heat treatment: crystallization, interlayer/interface healing, porosity evolution, and residual stress relaxation. Post-treatment mechanical testing revealed that interlaminar shear strength exhibits the largest and most consistent improvement, while tensile and flexural performance respond differently depending on material systems and treatment conditions. Thermal and dimensional results are shown to be mechanistically coupled between heat deflection maximization temperature and shrinkage or warping minimization. Reinforcement type and matrix chemistry play a substantial role in modulating the treatment response, as continuous-fiber and carbon-fiber systems generally benefit most from pressure-assisted treatment. The present review concludes by identifying persistent gaps in protocol standardization, interlaminar or fatigue characterization, and reinforcement-treatment coupling studies, while outlining directions, including in situ consolidation and data-driven process-property modeling for future work.

## 1. Introduction

Additive manufacturing (AM) of polymer matrix composites (PMCs) has transitioned over the past decade from a prototyping technology to a viable route for producing structural and semi-structural components across aerospace, automotive, biomedical, and defense sectors [1,2]. Processes such as fused filament fabrication (FFF) with short- and continuous-fiber reinforcement, vat photopolymerization (VPP) of fiber-filled resins, and powder bed fusion (PBF) of composite feedstocks now allow the layer-wise deposition of complex geometries that would be difficult or impossible to achieve through conventional molding or autoclave processing. This design freedom, however, comes at the cost of a microstructure that is fundamentally different from and sometimes inferior to that of traditionally manufactured composites, owing to the layer-by-layer thermal history intrinsic to AM [3].

The mechanical performance of AM-PMCs is governed primarily by phenomena that occur at and between deposited layers. These include incomplete interlayer diffusion and neck growth, voids trapped at raster boundaries, non-uniform fiber orientation and wetting, and residual stresses generated by repeated, spatially non-uniform heating and cooling cycles [4,5]. In semicrystalline thermoplastics such as PEEK, PEKK, PPS, and PA, the rapid cooling rates typical of extrusion-based AM further suppress crystallization, yielding parts with low and spatially heterogeneous crystallinity relative to their compression-molded or injection-molded counterparts [6,7]. For thermoset-based systems processed via VPP, the as-printed state is typically an incompletely cured network with a substantial fraction of unreacted functional groups [8]. In both thermoplastic and thermoset systems, the as-fabricated part represents a metastable or non-equilibrium state whose properties can be deliberately modified through post-processing.

Heat treatment has emerged as one of the most widely investigated post-processing strategies to address these limitations. By supplying thermal energy after fabrication, through annealing, confined or constrained annealing, autoclave post-consolidation, or controlled post-curing, it becomes possible to relax residual stresses, promote further crystallization or curing, enhance interlayer diffusion and healing, and, in some cases, reduce porosity [9,10,11]. Different studies have concluded that these mechanisms are not independent. Therefore, a treatment temperature and dwell time sufficient to increase crystallinity may simultaneously relieve residual stress, while also risking warping, fiber-matrix debonding, or thermo-oxidative degradation of the matrix. The overall effect of heat treatment on mechanical, thermal, and dimensional performance is therefore highly dependent on the interaction between material system, AM process, and treatment parameters. Consequently, to date, the literature reports a wide, and sometimes contradictory, range of outcomes [12,13,14].

Despite the growing number of published studies, several factors hinder the synthesis of a coherent and generalizable understanding of the effect of heat treatment in AM-PMCs. First, treatment protocols and conditions, such as temperature, dwell time, heating/cooling rate, use of constraining fixtures, and atmosphere, are not usually standardized across studies, which makes a direct comparison difficult [15]. Second, most investigations report bulk or intralaminar mechanical properties, while few studies analyze interlaminar behavior, even though interlayer bonding is the dominant failure-limiting feature of AM parts [16]. Third, the coupling between reinforcement type (short fiber, continuous fiber, particulate) and matrix response to heat treatment is inconsistently addressed, limiting the ability to draw conclusions that can be generalized across material systems [17]. Finally, long-term or cyclic loading behavior (fatigue, creep) after heat treatment remains comparatively underexplored in relation to quasi-static characterization.

Therefore, this review aims to consolidate and critically evaluate the current understanding of how post-fabrication heat treatment affects the microstructure and mechanical performance of additively manufactured PMCs. Specifically, it (i) classifies the different heat treatment strategies reported in diverse AM processes and material systems, (ii) examines and evaluates the underlying physicochemical mechanisms: crystallization, stress relaxation, interlayer diffusion, and porosity evolution, which are activated by thermal post-processing, (iii) effectively synthesizes the reported effects on mechanical, thermal, and dimensional properties, drawing particular attention to intralaminar versus interlaminar behavior, and (iv) identifies methodological inconsistencies and open research gaps that limit the application of heat-treated AM-PMCs in structural applications.

The remainder of this article is organized as follows: the methodology adopted to search and classify the most relevant articles is presented in Section 2; Section 3 describes the AM processes relevant to PMC fabrication and their characteristic as-printed microstructures; Section 4 classifies heat treatment methods and process parameters reported in the literature; Section 5, Section 6 and Section 7 examine, respectively, microstructural, mechanical, and thermal/dimensional effects of heat treatment; Section 8 presents a comparative analysis across reinforcement types and matrix systems; Section 9 discusses current challenges and research gaps; and Section 10 outlines future perspectives before Section 11 presents the concluding remarks.

## 2. Materials and Methods

**Figure 1:**
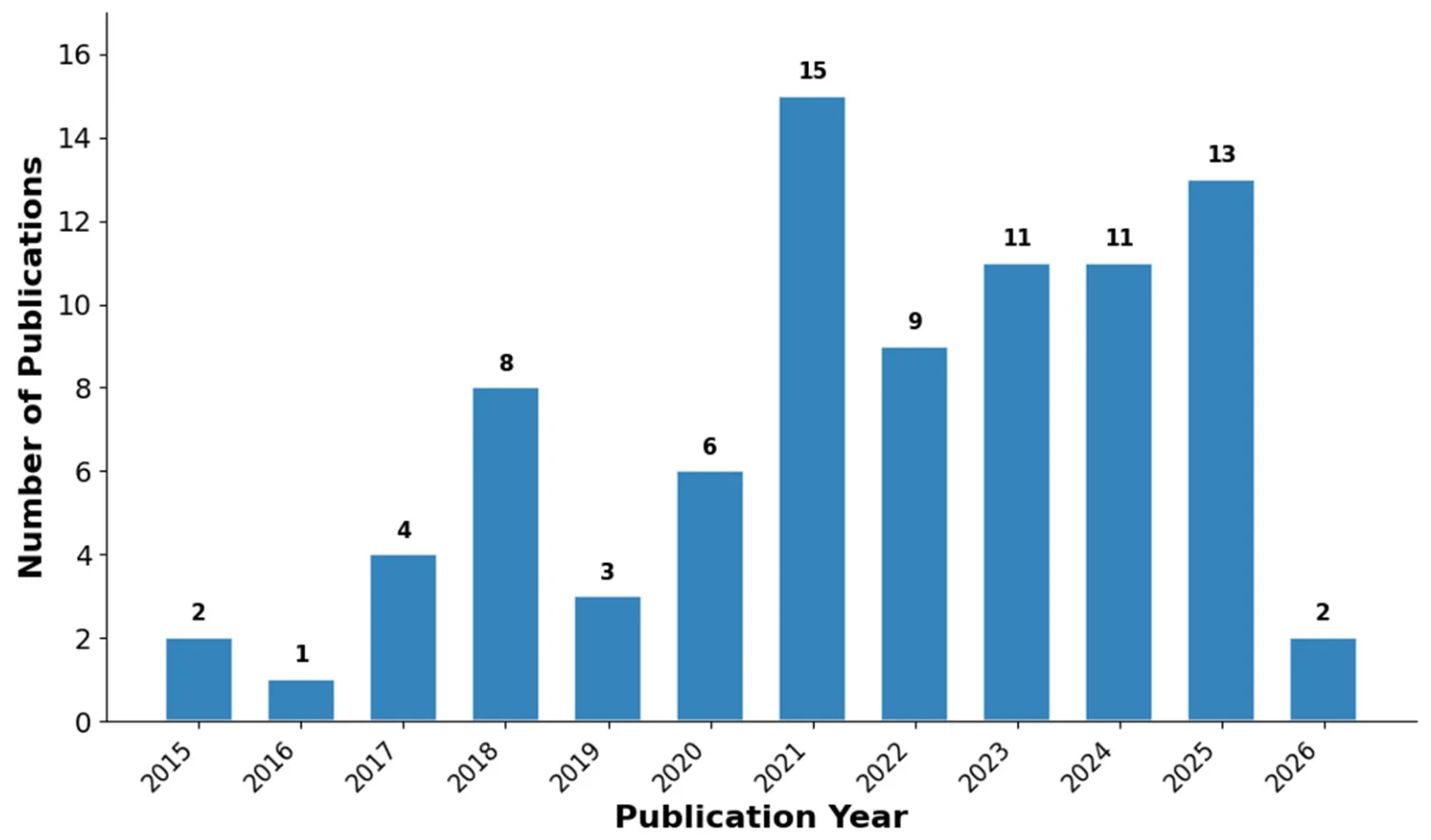
Published articles in the last decade focused on additive manufacturing, polymer matrix composites, and heat treatment.

The literature search for this review employed the keywords “additive manufacturing,” “polymer matrix composites,” and “heat treatment,” combined with the Boolean operator AND. The search identified 85 documents indexed in the Web of Science database (Figure 1), with the final search performed on 15 August 2026. The temporal distribution of publications reveals a progressive increase in research activity focused mainly on material extrusion processes.

Because this review focuses on PMCs, neat-polymer data are cited only where they provide a necessary baseline or contrast (e.g., matrix-only thermal stability or fiber-reinforcement effect size) and are explicitly identified as such throughout; unlabeled data should be read as composite systems.

Figures were created using Python (v3.14.7) and commonly available libraries, including ReportLab and Pillow. OpenAI Codex (GPT-5, accessed September 2026) was used as a coding assistant to draft, refine, and troubleshoot the Python code based on prompts specifying the authors’ concepts, scientific content, layouts, labels, dimensions, colors, and export formats. All figure concepts, design decisions, and final revisions were developed and approved by the authors.

## 3. Additive Manufacturing Processes for PMCs: Resulting Microstructures

**Figure 2:**
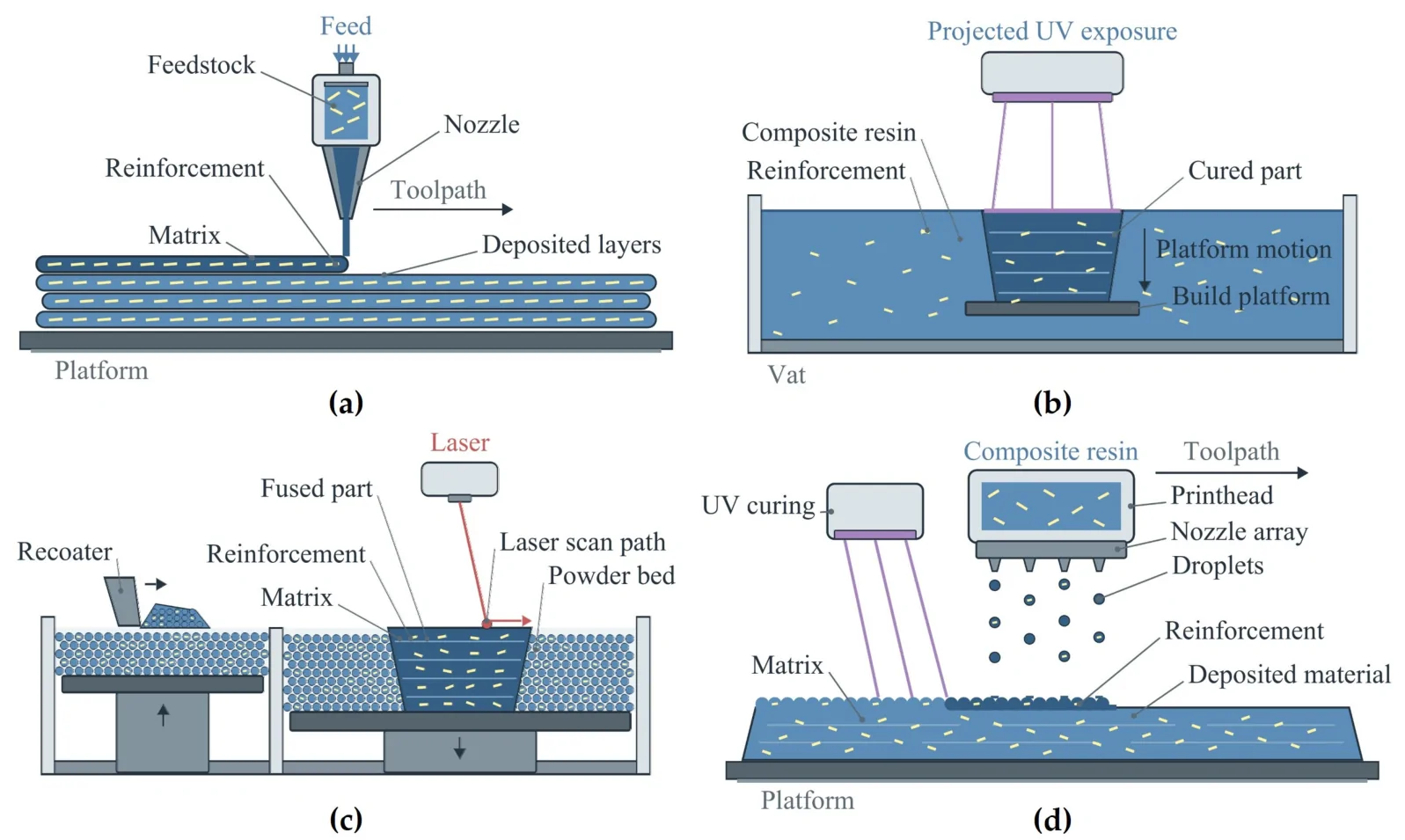
Additive manufacturing processes for PMCs: (**a**) material extrusion, including FFF/FDM and DIW, (**b**) vat photopolymerization (VPP), (**c**) powder bed fusion; and (**d**) material jetting.

The as-printed microstructure of an AM-PMC and therefore its response to subsequent heat treatment is dictated primarily by the deposition/consolidation physics of the process used to fabricate it. Four process families dominate the current literature (Figure 2): material extrusion, including fused filament fabrication or fused deposition modeling (FFF/FDM) and direct ink writing (DIW), vat photopolymerization (VPP), including stereolithography (SLA) and digital light processing (DLP), and finally powder bed fusion, mainly selective laser sintering (SLS), each producing a distinct starting point for thermal post-processing.

### 3.1. Material Extrusion

Material extrusion includes several nozzle-based AM processes, most notably FFF/FDM and DIW (Figure 2a). Although both processes deposit material along prescribed paths, they differ substantially in feedstock form, rheological requirements, solidification mechanisms, and thermal post-processing needs. They are therefore treated here as separate subcategories of material extrusion.

#### 3.1.1. Fused Filament Fabrication

Material extrusion (FFF/FDM) is the most extensively studied AM route for PMCs, owing to its low cost, compatibility with high-performance thermoplastics, and ability to process both short-fiber and continuous-fiber feedstocks [18,19]. In short-fiber systems, chopped fiber (carbon, glass, or natural fiber such as wood flour) is compounded into a filament and extruded through a heated nozzle. The resulting part exhibits fiber alignment predominantly along the raster direction, producing pronounced in-plane/out-of-plane (X-Y vs. Z) anisotropy [20]. In continuous-fiber systems (e.g., Markforged-type platforms), a dry or pre-impregnated fiber tow is co-deposited with a thermoplastic matrix, which improves stiffness and strength along the fiber path but introduces additional risk of poor fiber wetting when contact time between molten polymer and fiber in the nozzle is insufficient [1,2].

Regardless of fiber format, the governing microstructural feature of FFF-printed parts is the discontinuous, layer-by-layer deposition sequence itself. Each new bead is deposited onto a previously cooled layer, so bonding occurs through localized reheating, wetting, and polymer chain interdiffusion across the bead interface, a process that is frequently incomplete under standard processing conditions and leaves voids concentrated at raster and interlayer boundaries [21]. For semicrystalline high-performance matrices, such as PEEK, PEKK, and PPS, the same rapid and uneven cooling process that limits interlayer diffusion also restricts crystallization. FFF-printed specimens typically exhibit a crystallinity between 18 and 35%. In contrast, to the overall higher crystallinity of around 30–48%, achievable through compression-molded PEEK, given that crystallinity is strongly sensitive to nozzle temperature, chamber/bed temperature, and layer cooling rate [22]. Given that crystallinity governs matrix modulus and load transfer efficiency across the interlayer interface, the as-printed specimen usually exhibits a comparatively low interlaminar strength, which is commonly the weakest mechanical mode in FFF composites [23].

#### 3.1.2. Direct Ink Writing

Direct ink writing (DIW), in contrast to FFF, deposits a viscoelastic polymer or composite ink through a nozzle, typically at ambient or moderately elevated temperature (<250 °C) [24]. The feedstock is generally a shear-thinning paste, gel, or resin formulation that flows under pressure and rapidly recovers sufficient stiffness after extrusion, allowing the deposited material to retain its shape as a green structure [25]. In polymer matrix composites, DIW inks may contain short fibers, particles, or other reinforcements that become preferentially aligned along the deposition direction because of shear flow through the nozzle [26,27].

Final consolidation may occur through thermal or ultraviolet curing, solvent evaporation, drying, gelation, or another physical or chemical mechanism. Consequently, the as-deposited microstructure is governed primarily by ink rheology, reinforcement alignment, interfilamentous bonding, and curing- or drying-induced shrinkage rather than by melt crystallization kinetics. In thermoset-based DIW, thermal treatment may provide either the primary cure or a subsequent post-cure, increasing polymer conversion, crosslink density, glass-transition temperature, and mechanical performance. However, inappropriate curing conditions may also promote shrinkage, warping, residual stresses, or incomplete bonding between adjacent filaments [28,29].

### 3.2. Vat Photopolymerization

Vat photopolymerization (VPP) processes (Figure 2b) cure a liquid photopolymer resin layer-by-layer using UV or visible light, and have more recently been extended to particulate- and fiber-filled resins by dispersing milled or chopped fibers, or by interleaving continuous fiber fabric between cured resin layers [30]. Because light must penetrate the resin to initiate polymerization, filler content, particle size, and refractive index mismatch between filler and resin strongly affect cure depth and cure uniformity, and the light-scattering behavior of embedded fibers can leave the resin incompletely cured in filler-rich regions relative to the free surface [31], in reference to filled photopolymer curing. Layer-by-layer curing in VPP composites has been reported to produce a comparatively low intrinsic void content relative to extrusion-based processes when fiber content is low, since each layer polymerizes as a continuous film rather than as discrete deposited beads. However, void content rises with reinforcement volume fraction and layer count as trapped air and incomplete resin infiltration around fibers become more likely, as reported by Nugraha et al. [32], for multilayer glass-fiber VPP composites. More broadly, in frontally or thermally cured thermoset composites, degree of cure values on the order of 70–85% have been reported immediately after processing, substantially below the near-complete conversion achieved in autoclave-cured references, leaving residual reactive functional groups available for post-curing on frontal-polymerization carbon/epoxy composites [33].

### 3.3. Powder Bed Fusion Techniques

Selective laser sintering (SLS) of polymer composites (commonly glass- or carbon-filled polyamide) consolidates powder particles through localized laser-induced melting within a heated powder bed (Figure 2c); the elevated bed temperature promotes more uniform interparticle fusion than extrusion-based processes but leaves characteristic interparticle porosity where sintering necks fail to fully coalesce, particularly around filler particles that locally disrupt melt flow [34]. Microstructural heterogeneity tied to powder thermal history, with virgin-polymer domains coexisting alongside recycled/aged powder domains (greater chain-scission degradation, lower molecular weight), introduces batch-to-batch variability with no direct equivalent in FFF or VPP.

### 3.4. Material Jetting

Material jetting (MJ) selectively deposits droplets of a low-viscosity polymer or composite feedstock through one or more printheads, followed by rapid solidification, most commonly through ultraviolet photopolymerization (Figure 2d). Unlike vat photopolymerization, in which selected regions of a resin bath are cured, MJ places the build material only where it is required and can deposit multiple materials within the same layer. Polymer matrix composites produced by MJ typically contain particles, nanoparticles, or low concentrations of short fibers because increasing reinforcement content raises viscosity, promotes sedimentation, disrupts droplet formation, and increases the risk of nozzle clogging [35,36]. Reinforcements may also scatter or absorb the curing light, producing spatial variations in conversion and crosslink density.

The as-printed microstructure of material-jetted composites is therefore governed by droplet coalescence, interlayer bonding, filler dispersion, and the extent of photopolymerization [37]. Subsequent ultraviolet or thermal post-curing can increase polymer conversion, crosslink density, glass-transition temperature, stiffness, and dimensional stability [38,39]. However, increasing the duration of the curing process may also increase shrinkage, residual stresses, warping, or brittleness, especially when aggressive cure gradients or a thermal expansion mismatch exist between the matrix and the reinforcement. Although MJ is a less popular fabrication method for structural PMCs than material extrusion or powder bed fusion, it is still positioned as a relevant alternative for thermal post-treatment because its response is closely linked to incomplete initial curing or the presence of filler-induced heterogeneity.

### 3.5. Common Microstructural Signature Across Processes

Despite their differing physics, the four process families converge on a common set of as-fabricated deficiencies relative to conventionally processed composites: (i) process-induced porosity concentrated at layer or bead interfaces, (ii) an incomplete degree of matrix consolidation, sub-equilibrium crystallinity in semicrystalline thermoplastics or sub-maximal conversion in thermosets, (iii) pronounced mechanical anisotropy arising from both fiber orientation and the directionality of interlayer bonding, and (iv) residual stresses generated by repeated, spatially non-uniform thermal cycling during build. Table 1 provides a concise comparison of the dominant as-printed defects across AM processes. It is precisely this combination of incomplete consolidation and residual stress that motivates the use of post-fabrication heat treatment, and the relative weight of each deficiency. Porosity-dominated in FFF, cure-limited in VPP thermosets, and diffusion-limited in SLS—determines which heat treatment mechanism (discussed in Section 4) is most relevant to a given material–process combination.

## 4. Heat Treatment Methods and Process Parameters: A Taxonomy

**Figure 3:**
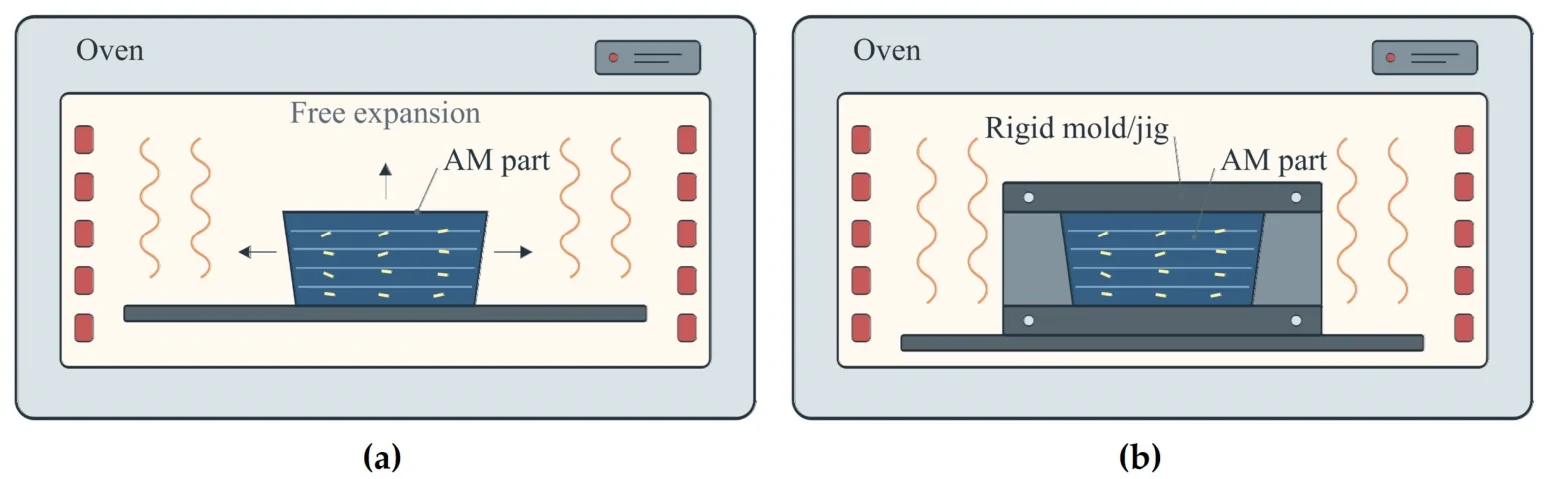
Thermal annealing processing for PMCs: (**a**) unconstrained, (**b**) constrained.

The heat treatments reported for AM-PMCs are heterogeneous in both timing (applied after the part is complete, or during layer deposition), in the physical fields imposed (heat only, heat plus pressure, or heat combined with actinic radiation), and in the deficiency they target (sub-equilibrium crystallinity and locked-in stress in solidified thermoplastics; residual porosity and weak interlayer welds in fiber-reinforced thermoplastics; incomplete monomer conversion in thermosets and photopolymers). To make these distinctions explicit, the treatments reported to date are organized here into four mechanistically separate categories: (i) post-print thermal treatment, encompassing free and constrained annealing of solidified thermoplastic-composite parts, in which heat alone drives stress relaxation, secondary crystallization, and limited interbead diffusion (Figure 3, Section 4.1); (ii) thermo-mechanical consolidation, encompassing hot isostatic pressing (HIP), silicone-mold HIP, and uniaxial hot pressing of solidified fiber-reinforced thermoplastic composites, in which applied pressure drives void closure and interlayer autohesion concurrently with thermally activated interdiffusion (Section 4.2); (iii) post-curing, encompassing thermal, UV, combined UV/thermal, and electron-beam post-treatments of photopolymer- and thermoset-based AM parts, in which the mechanism activated is the completion of the monomer-to-network conversion rather than any melt-mediated process (Section 4.3); and (iv) in situ thermal processing, encompassing in-nozzle infrared preheating, integrated roller compaction, and other localized heat- and/or pressure-application strategies that act on the freshly deposited road during the build itself rather than on the finished part (Section 4.4).

Section 5 then discusses the microstructural mechanisms—crystallization, interface healing, porosity evolution, and residual-stress relaxation—that these four treatment categories activate in different proportions.

### 4.1. Post-Print Thermal Treatment (Annealing)

#### 4.1.1. Free (Unconstrained) Thermal Annealing

Unconstrained annealing consists of heating a printed part in an oven or heated medium above a target temperature, kept for a defined dwell time, and subsequently cooled without applying any external mechanical restraints (Figure 3a). This method is the most widely reported treatment for thermoplastic-composite AM parts [40]. Different studies report a wide range of parameter setups and treatment temperatures, which are typically referenced to the matrix glass transition (*Tg*) or melting temperature (*Tm*), ranging from sub-*Tg* conditions destined for stress relaxation (e.g., 90–130 °C for PETG-fiber composites, with dwell times from 30 to 480 min) [40] to near-*Tm* conditions intended to promote recrystallization in semicrystalline matrices such as PLA, PA, and PEEK. Reported outcomes generally depend on the process and material. For instance, annealing has been observed to effectively increase tensile and flexural strength, hardness, and interlaminar toughness by reducing residual stress, increasing crystallinity, and improving interbead bonding [40,41]. Furthermore, several studies conclude that annealing can reduce fracture toughness while increasing porosity due to residual moisture, volatiles, or entrapped gas expansion during heating [40,41]. The heating medium itself has also been varied: comparative studies of oven annealing versus sand-bath annealing report that the more gradual heating and cooling imposed by a granular medium reduces thermal shock and dimensional distortion relative to conventional oven annealing, at the cost of longer processing times [42].

#### 4.1.2. Constrained (Confined) Annealing

Constrained annealing applies the same thermal cycle as free annealing but restrains the part, typically within a rigid or semi-rigid mold, jig, or fixture during heating, to suppress the warping and dimensional expansion that unconstrained parts exhibit when residual stresses relax above *Tg* [10]. This approach is reported particularly for parts where dimensional tolerance is critical, since unconstrained annealing above *Tg* can produce measurable expansion perpendicular to the print layers as locked-in residual stresses are released [12]. Constrained annealing is mechanistically similar to that in Section 4.1 but trades some of the stress-relaxation benefit for dimensional control, since full stress release is inherently coupled to a shape change that the constraint is designed to prevent (Figure 3b).

### 4.2. Thermo-Mechanical Consolidation

Where free or constrained annealing alone is insufficient to close process-induced voids, pressure-assisted treatments combine elevated temperature with applied isostatic or uniaxial pressure to mechanically collapse porosity and improve fiber-matrix and interlayer contact. Hot isostatic pressing (HIP), adapted from its established use in metal AM [43,44], has been applied to continuous-fiber thermoplastic composites by heating above *Tg* (but typically below or near *Tm* to avoid excessive fiber-bed disruption) under gas or silicone-mold-transmitted pressures on the order of 1–15 MPa (10–150 bar) [45]. Reported effects include a decreased porosity ranging from roughly half to near-elimination, and the enhancement of interlaminar shear strength (ILSS) from approximately 30% to more than an order of magnitude when compared to as-printed material, depending on treatment temperature, pressure, and starting void content [46]. HIP for polymer matrices differs from HIP for metal matrices due to the possibility of equilibrating gas-based isostatic pressure through interconnected as-printed porosity prior to its collapse. For this reason, different approaches have been designed to use silicone molds to keep a differential pressure across the surface of the specimen to allow pore closure [46]. Related uniaxial hot-pressing and roller-compaction variants, including in-process compaction rollers integrated at the print nozzle, achieve similar densification objectives with simpler tooling, at the cost of being largely restricted to flat or simple geometries [47].

### 4.3. Post-Curing (Thermoset and Photopolymer Systems)

For thermoset-based DIW, VPP, MJ, and other photopolymer-based AM processes, the principal post-fabrication thermal treatment is post-curing rather than annealing, since the as-printed part is limited by incomplete monomer conversion rather than by sub-equilibrium crystallinity. For example, Lewicki et al. [26] developed a carbon-fiber/epoxy DIW system based on latent thermal curing, for which a post-cure at 80 °C for 12 h was reported to achieve full network density, while substantially faster curing was possible at higher temperatures. Reported post-cure protocols include UV-only exposure, thermal-only exposure (typically 50–250 °C for 10 min to over an hour under inert atmosphere), combined UV-plus-heat exposure, and, less commonly, electron-beam post-curing [48]. Across these variants, post-cure temperature and time consistently increase degree of conversion and tensile strength, but the relative effectiveness of UV versus thermal post-cure has been reported to be resin-chemistry-dependent; thermal post-curing is not always as effective as UV post-curing for a given acrylate or thiol-ene formulation [30,48] and prolonged or high-temperature post-cure has also been associated with yellowing/discoloration and, in dental and biomedical resins, altered biocompatibility profiles that must be balanced against the mechanical benefit [30,48]. The main advantages and limitations of UV-only and thermal-only post-curing are detailed below.

UV-only post-curing:

*Advantages*: Rapid and localized curing; effective for increasing conversion and crosslink density in photopolymer systems.

*Limitations*: Limited light penetration in thick or highly filled/reinforced parts; light scattering or absorption can produce heterogeneous conversion; excessive exposure may cause discoloration.

Thermal-only post-curing:

*Advantages*: More uniform treatment of bulk components; can promote further conversion in systems where thermal curing is dominant or where UV penetration is limited.

*Limitations*: Generally, requires longer treatment times and elevated temperatures; excessive temperature or exposure can promote discoloration, embrittlement, shrinkage, or other thermal effects.

### 4.4. In Situ Thermal Processing

The three treatment categories above (Section 4.1, Section 4.2 and Section 4.3) are applied to a part that has already been fully deposited and solidified. A conceptually distinct family of strategies instead applies heat, or heat combined with mechanical compaction, during the deposition step itself, intending to activate some of the same mechanisms—interlayer diffusion, void closure, and residual-stress reduction—before the interface has fully vitrified, and thereby reducing or eliminating the need for a subsequent post-print treatment.

Reported implementations include in-nozzle infrared preheating of the substrate immediately ahead of the deposition point, in which a focused IR source raises the temperature of the previously deposited road above *Tg* (or, for semicrystalline matrices, into the crystallization window) at the moment a new material is placed on top; integrated roller or wheel compaction at the print head, in which a mechanical roller travels immediately behind the nozzle and applies a controlled normal force to the freshly deposited bead; and combined strategies in which conformal IR preheating and dual-roller hot pressing are integrated into the same continuous-fiber deposition head. In one such implementation for continuous carbon-fiber/PEEK printing, this combined in-process approach was reported to increase interlaminar shear strength by approximately 117% at optimized preheating temperature and layer height [49]. In situ thermal processing does not, in the studies reviewed here, fully replace post-print treatment for high-performance semicrystalline matrices such as PEEK, in which post-print annealing is still required to reach target crystallinity, but it does shift a meaningful fraction of the interlayer-healing benefit upstream and is discussed further in Section 10 as an emerging direction for reducing the energy and time cost of post-processing.

### 4.5. Summary Taxonomy

Table 2 summarizes the four treatment families discussed above according to the material class they primarily address, the mechanism activated, and the principal trade-off reported in the literature.

This taxonomy provides the organizing structure for Section 5, where the underlying physicochemical mechanisms common to several of these treatment families, crystallization, stress relaxation, interlayer diffusion, and void evolution, are examined in more mechanistic detail.

## 5. Effects on Microstructure: Crystallinity, Porosity, Interface, and Residual Stress

**Figure 4:**
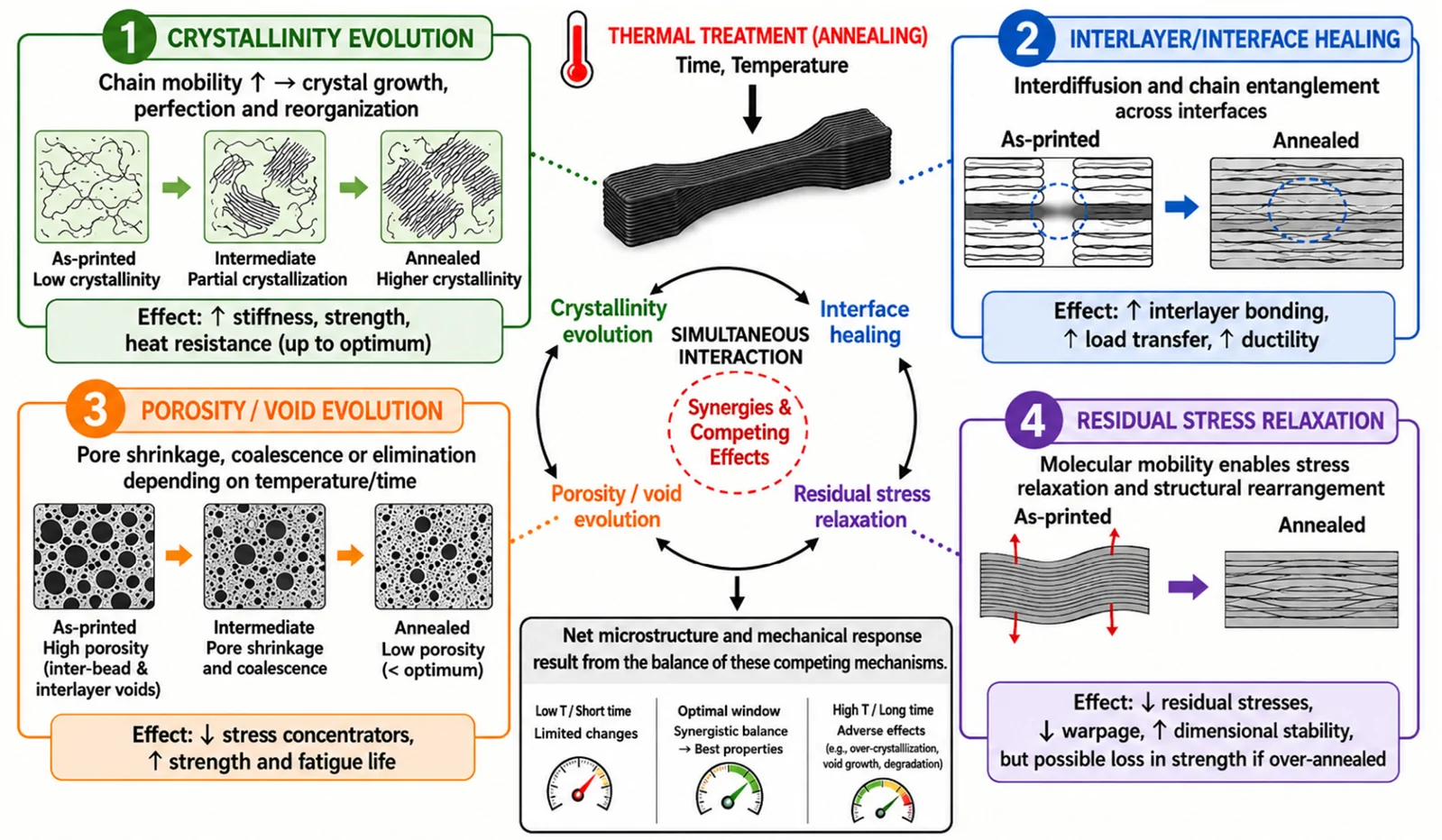
Schematic illustration of the four interrelated microstructural mechanisms governing the evolution of additively manufactured PMCs during thermal annealing: (1) crystallinity evolution through enhanced molecular-chain mobility and crystalline reorganization, (2) interlayer/interface healing driven by polymer-chain diffusion and entanglement across adjacent deposition roads, (3) porosity and void evolution through pore shrinkage, coalescence, or elimination, and (4) residual stress relaxation through molecular rearrangement.

The mechanical and thermal improvements attributed to heat treatment in Section 4 arise from four interrelated microstructural mechanisms, depicted in Figure 4: crystallinity evolution, interlayer/interface healing, porosity/void evolution, and residual stress relaxation. These mechanisms occur simultaneously and may exhibit synergistic or competing effects depending on annealing temperature and duration, ultimately determining the resulting microstructure and mechanical response.

It’s worth noting that the strength of experimental evidence supporting these mechanisms varies across the literature (e.g., crystallinity by DSC/XRD, porosity by SEM/µCT, residual stress by hole-drilling or XRD sin(^2^ψ)). Where studies directly characterize crystallinity, interlayer morphology, porosity, or residual stress, the corresponding mechanism can be considered experimentally demonstrated. In other cases, mechanisms are inferred from changes in mechanical or thermal properties and should therefore be interpreted as plausible explanations rather than directly verified causal relationships.

### 5.1. Crystallinity Evolution

In semicrystalline matrices (PLA, PA, PP, PEEK, PEKK, PPS), the rapid, non-isothermal cooling intrinsic to layer-wise deposition produces an as-printed crystallinity well below the equilibrium value attainable under slow, isothermal cooling, as noted in Section 3 [23]. Annealing above *Tg* but below *Tm* provides the chain mobility required for secondary crystallization to approach this equilibrium value, and the resulting increase in the crystalline fraction raises the matrix modulus and strength because crystalline lamellae act as physical crosslinks and reinforcing domains within the amorphous matrix [40]. Reported improvements attributed at least in part to increased crystallinity include increases in tensile strength and Young’s modulus in FFF PLA and PEEK composites [8], and a roughly 20% increase in hardness in annealed PETG-based composites [64]. Crystallization during annealing is not, however, unconditionally beneficial: because crystalline regions are denser than the amorphous phase, densification during crystallization drives shrinkage, and where this shrinkage is spatially non-uniform (e.g., higher fiber content locally restricting matrix crystallization), it can introduce new internal stress rather than only relieving the original printing-induced stress. In nylon (PA) systems specifically, the crystalline morphology developed during the brief, non-isothermal thermal history at the weld zone, rather than bulk crystallinity alone, has been identified as a controlling factor: slower crystal growth at the interlayer weld is associated with lower tie-chain density in the interlamellar amorphous regions, which reduces interlayer bond strength independently of overall part crystallinity [21].

### 5.2. Interface Healing and Interlayer Diffusion

Interlayer (and, in multi-fiber-tow systems, inter-tow) bonding is generally the mechanically weakest feature of an AM-PMC, since each new bead or layer is deposited onto a partially cooled surface, sharply limiting the time available for polymer chain interdiffusion before the interface vitrifies. The governing physical framework for this process is de Gennes’ reptation theory [65], in which polymer chains diffuse across the interface as a function of temperature, contact time, and molecular weight, combined with Wool and O’Connor’s healing model [66], in which interfacial strength develops proportionally to the interpenetration depth raised to a characteristic power (commonly reported as a 1/4-power time dependence for shear strength and a 1/2-power dependence for fracture toughness) until a critical interpenetration depth restores bulk-material strength [67]. Under the brief, non-isothermal thermal history typical of as-printed FFF, this critical interpenetration depth is rarely reached, leaving the interface as a mechanically distinct, under-healed zone [67,68]. Post-print annealing re-supplies the temperature and time needed to continue this interdiffusion process beyond what was achieved during printing, and reported bending-strength recovery after annealing has been attributed specifically to enhanced intermolecular diffusion and entanglement density above *Tg*, with the presence of short fibers further influencing (and in some reports, boosting) this healing response by locally altering matrix mobility near the fiber-matrix boundary [41,69]. Because healing kinetics depend on contact pressure as well as temperature and time, pressure-assisted treatments (Section 4.3) address the interface mechanism directly by both promoting interdiffusion and mechanically closing any residual gap at the weld line, which is consistent with the larger ILSS gains reported for HIP/hot-pressing relative to free annealing in several studies [68].

### 5.3. Porosity and Void Evolution

Two physically distinct populations of voids are typically distinguished in AM-PMCs: intrinsic process voids, formed by incomplete wetting or entrapped air at raster and interlayer boundaries during deposition, and secondary voids that can form or grow during heat treatment itself, from residual moisture, entrapped volatiles, or gas expansion as the part is heated [23]. The response to heat treatment of the first void population is generally favorable, as elevated temperatures and longer dwell times enhance matrix flow, which tends to fill intrinsic voids, while pressure-assisted treatments compress the specimens, allowing these types of voids to mechanically collapse. Through this process, porosity reductions can range from approximately 50% to an almost complete densification depending on the initial void fraction, applied pressure, and treatment conditions [45,60]. On the other hand, the response of the second void population can be disadvantageous, as several studies report a reduction of the fracture toughness and an enhancement of void content after free (unconstrained, non-pressurized) annealing, which is usually attributed to gas or moisture expansion within the specimen during heating without a mechanism that allows its exhaustion [70]. This distinction explains an apparent contradiction in the literature, that annealing simultaneously “removes” and “creates” porosity depending on the study, and underscores that treatment atmosphere, prior moisture conditioning, and the presence/absence of applied pressure are as consequential as treatment temperature and time in determining the net porosity outcome.

### 5.4. Residual Stress Relaxation

Residual stresses in AM-PMCs originate from the repeated, spatially non-uniform thermal cycling each layer experiences as subsequent layers are deposited on top of each other, compounded in fiber-filled systems by the coefficient-of-thermal-expansion mismatch between fiber and matrix [71]. Therefore, heating the as-printed specimens above *Tg* increases polymer chain mobility up to a threshold at which these locked-in stresses can relax, which is consistently reported as a contributing mechanism to the improvement of the mechanical response of the parts after annealing [40]. However, stress relaxation is mechanically tied to a macroscopic change in the shape of the specimen because the residual stress field suppresses both warping and out-of-plane expansion in the as-printed state. Consequently, specimen relaxation during unconstrained annealing is frequently accompanied by measurable dimensional expansion perpendicular to the print layers, which can lead to visible warping or distortion in asymmetric or geometrically complex parts. This is the direct motivation for constrained annealing (Section 4.2): by physically restraining the part during the temperature excursion that enables stress relaxation, dimensional stability is partially preserved, at the cost of incomplete stress relief relative to a fully unconstrained treatment.

### 5.5. Coupling Between Mechanisms

The four aforementioned mechanisms are not independent, and their relative dominance, rather than any single one in isolation, determines the net effect of a given heat treatment on final properties. Crystallization and stress relaxation are thermally activated by the same temperature excursion and proceed concurrently, but crystallization-driven densification and stress-relief-driven expansion act in geometrically opposing directions, which can partially cancel or, in spatially non-uniform parts (e.g., regions of varying local fiber content or layer thickness), generate new internal stress gradients even as the original printing-induced stress is relieved [12]. Interface healing and the collapse of porosity are both promoted by the same temperature and time window that increases crystallization. However, healing is mostly dependent on the applied contact pressure. For this reason, pressure-assisted methods improve interlaminar rather than in-plane properties [60]. Finally, the addition of different fiber reinforcements influences all four thermally driven mechanisms simultaneously by promoting a local restriction of crystallization kinetics, modifying interfacial chain mobility, and acting as a barrier to void closure. Consequently, the net microstructural outcome of a nominally identical heat treatment can differ substantially between an unreinforced matrix and its fiber-filled counterpart, a dependency examined further in Section 8.

## 6. Effects on Mechanical Performance

**Figure 5:**
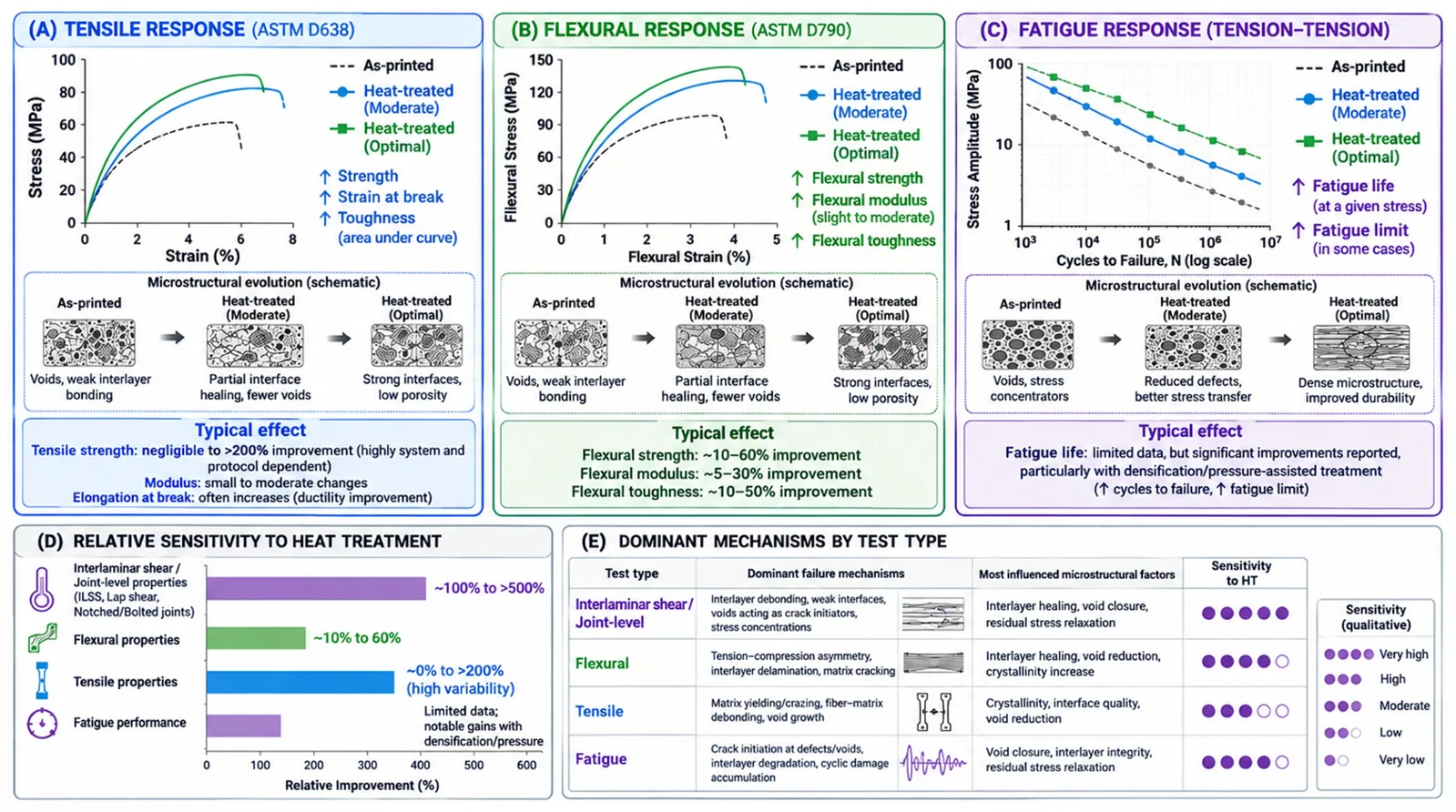
Representative evaluation of the mechanical response of AM-PMC: (**A**) Tensile, (**B**) flexural, (**C**) fatigue, (**D**) property response as a function of heat treatment, (**E**) failure mechanisms.

This section synthesizes reported heat treatment effects by mechanical test type, since the magnitude and occasionally the sign (+/−) of the effect differ substantially between in-plane (tensile, flexural, compressive, impact) and interlaminar/joint-level (short-beam shear, lap shear, notched/bolted-joint) loading modes, as well as under cyclic loading (Figure 5).

Reported ILSS improvements span 100–500% or more, and tensile gains exceeding 200% have been documented; these ranges reflect substantial heterogeneity in reinforcement type and fraction, print orientation, heat-treatment protocol, and test method across the cited studies, and should be read as an indication of the potential magnitude of improvement under favorable conditions rather than a directly comparable, method-controlled effect size.

### 6.1. Tensile Properties

Tensile strength and modulus improvements after annealing are the most consistently reported outcome across matrix systems, though magnitudes vary widely with material, temperature, and dwell time. For PLA and PETG composites, reported gains include a two- to three-fold increase in tensile strength for short-carbon-fiber-reinforced PLA and PETG after annealing [41], and improvements on the order of 8–25% in tensile strength for annealed carbon-fiber PETG relative to as-printed material, alongside comparable gains in hardness, impact strength, and bending strength. In carbon-fiber-reinforced polyamide (PAHT-CF), annealing at a manufacturer-recommended condition (80 °C, 12 h) produced a 50% increase in room-temperature tensile strength accompanied by a 70% reduction in strain at failure, with the strength advantage persisting (17.5%) even at elevated test temperatures up to 150 °C, indicating that annealing-induced improvements are not purely a room-temperature artifact [72]. Not all reported tensile effects are positive, however: prolonged annealing time has been associated with reduced tensile properties in neat PLA, attributed to stress build-up from continued, non-uniform crystallization or dimensional distortion during extended thermal exposure [73]. Yu et al. [40] reported that heat treatment can have minimal effect on tensile strength specifically, while more strongly affecting fracture behavior, underscoring that tensile strength alone is an incomplete indicator of treatment efficacy.

### 6.2. Flexural, Compressive, and Impact Properties

Flexural and compressive properties generally track the same direction of improvement as tensile properties but with distinct sensitivity to treatment temperature and time. In neat PLA, an annealing process at a temperature of 130 °C maintained for 300 s enhanced the flexural strength by approximately 58.3%, while a treatment at 140 °C for 600 s improved the sample’s compressive strength by around 39.8%; both enhancements were attributed to improved interlayer bonding [74]. Similarly, the optimization of neat PLA annealing conditions was performed through a Taguchi/ANOVA/Grey Relational Analysis (70–110 °C, 40–200 min), which identified that a treatment at a temperature of 90 °C for 120 min yielded the most balanced improvement in tensile, flexural, compressive, and impact strength, with treatment temperature serving as the most decisive factor compared to dwell time [75]. Impact strength improvements of similar relative magnitude (in the order of 5–25%) are reported alongside tensile and flexural gains in annealed PETG and carbon-fiber PETG [41], consistent with the interpretation that impact resistance benefits from the same interlayer-bonding and crystallinity mechanisms discussed in Section 5, though impact properties are also reported to be comparatively more sensitive to any annealing-induced porosity increase (Section 5.3) than quasi-static strength measures.

### 6.3. Interlaminar Shear and Joint-Level Properties

Interlaminar and joint-level properties show the largest reported relative improvements from heat treatment among all mechanical test types, consistent with Section 5.2’s identification of interlayer bonding as the primary microstructural target of thermal post-processing. In short- and continuous-fiber-reinforced PEEK, optimized heat treatment increased interlaminar shear strength (ILSS) from 5.78 to 39.05 MPa, more than a six-fold increase, through combined effects on porosity and interlayer diffusion [64]. In 3D-printed continuous carbon and aramid fiber composites with an Onyx matrix, ILSS increased steadily with exposure temperature up to 170 °C, peaking at 196% and 203% of baseline for CFRP and AFRP respectively, and remaining substantially elevated (173–183% of baseline) even at 200 °C, attributed to annealing-driven fiber-matrix reconsolidation; glass-fiber composites (GFRP) in the same study showed a markedly weaker and less consistent response, with ILSS falling to 84% of baseline at 200 °C due to thermal microcracking, indicating that fiber type modulates the interlaminar response to heat treatment independently of matrix chemistry [76]. Acoustic-emission-based damage analysis of heat-treated continuous-fiber composites reported maximum-load increases of 83.6% and 86.8% for specimens with 5% and 15% fiber volume fraction, respectively, after treatment at 180 °C, with the treatment shifting the dominant failure mode toward more matrix-dominated (rather than pure delamination) damage signatures [77]. Pressure-assisted interlayer treatments produce comparable or larger gains through the combined thermal-plus-mechanical mechanism discussed in Section 4.3 and Section 5.2: infrared preheating combined with dual-roller hot pressing during continuous carbon-fiber/PEEK printing increased ILSS by 117% at optimized preheating temperature and layer height [78]. At the joint level, post-print heat treatment has also been reported to restore interlayer tensile strength in carbon-fiber PETG to levels comparable to unreinforced PETG (which is otherwise reduced by fiber presence in the as-printed state) [79], and to improve tensile performance of open-hole and bolted-joint continuous carbon/glass fiber laminates relative to untreated equivalents, with glass-fiber joints retaining a higher fraction of unnotched strength than carbon-fiber joints regardless of treatment condition [80].

### 6.4. Fatigue Behavior

Fatigue performance after heat treatment remains comparatively underexplored. Evidence for pure thermal annealing is limited but indicates that heat treatment can improve fatigue strength, as reported for neat PLA [64], likely through enhanced interlayer healing and microstructural stabilization. In contrast, pressure-assisted consolidation combines thermal activation with mechanical densification and has demonstrated more pronounced fatigue endurance in continuous carbon-fiber thermoplastic composites. In Saeed et al.’s [81] study, heat-and-pressure-treated specimens survived beyond one million cycles at 80% UTS, whereas specimens tested at 90% UTS failed at approximately 50,000 cycles. Because these studies involve different materials and testing conditions, the available evidence does not permit a direct quantitative comparison between thermal and pressure-assisted treatments. Nevertheless, pressure-assisted consolidation appears particularly promising where fatigue performance is limited by interlayer porosity and incomplete consolidation. The fatigue-life improvement, despite comparatively modest tensile-strength change, is consistent with the fatigue-crack-initiation role typically played by interlayer voids and under-healed interfaces (Section 5.2 and Section 5.3), which cyclic loading is more sensitive to than a single monotonic pull to failure [82].

### 6.5. Synthesis Across Test Types

Ranking the relative sensitivity of mechanical test types to heat treatment, the literature surveyed here consistently places interlaminar shear and joint-level properties at the top (relative improvements from roughly 100% to over 500%), followed by flexural and impact properties (typically 10–60% improvement), tensile properties (highly variable, from negligible to 200%+ depending on matrix/fiber system and treatment protocol), and fatigue life (limited data, but improvements reported specifically where treatment includes a densification/pressure component). The temperature–time regimes at which these gains are reported, together with the associated crystallinity and dimensional response, are tabulated in Table 3. The ordering of the mentioned results supports the conclusion drawn in Section 5: interlayer healing and porosity closure are the dominant mechanisms by which the performance of AM-PMC is improved through heat treatments, as the most significant and consistent improvements are produced in properties governed by interlayer integrity (ILSS, fatigue), while smaller and treatment-condition-dependent improvements are obtained in properties governed by bulk matrix behavior, such as tensile modulus in well-consolidated and low-fiber-content materials.

## 7. Effects on Thermal and Dimensional Stability

Besides the mechanical performance analyzed in Section 6, heat treatment also modifies two other performance categories, which are relevant to service conditions: (*i*) thermal properties governing high-temperature usability—*Tg*, heat deflection temperature (HDT), thermal degradation onset—and (*ii*) dimensional stability—shrinkage, warping, and volumetric change. It is worth mentioning that dimensional stability is coupled to the residual-stress-relaxation mechanism discussed in Section 5.4.

**Figure 6:**
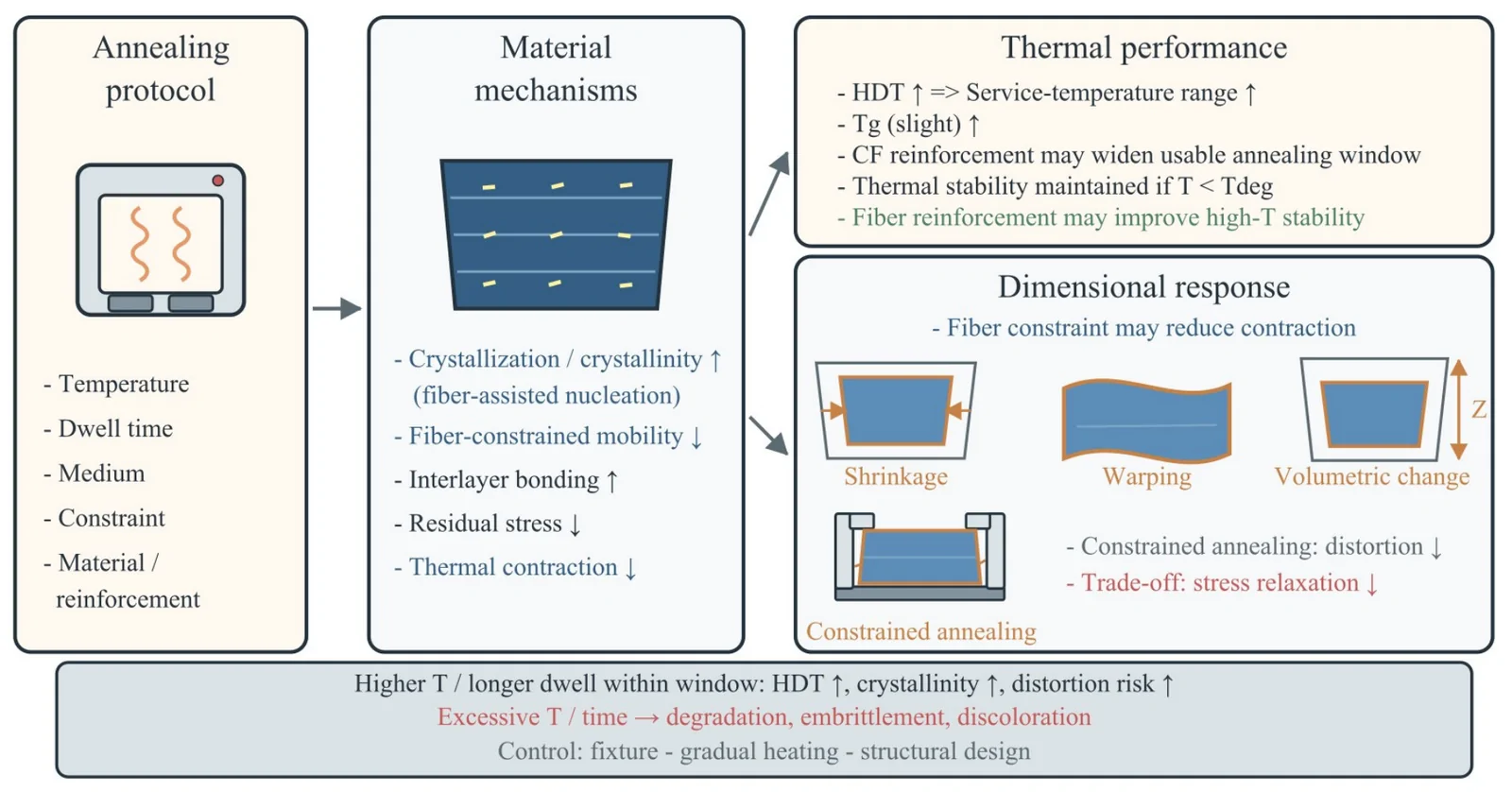
Schematic illustration of the effects on thermal and dimensional stability on PMCs.

Figure 6 illustrates the coupled effects of annealing protocols and material mechanisms with their respective thermal and dimensional responses in PMCs. The annealing protocol is determined by different conditions, such as temperature, dwell time, heating medium, geometric constraint, and material/reinforcement characteristics, which collectively regulate the sample crystallization, fiber-assisted nucleation, chain mobility, interlayer bonding, residual-stress relaxation, and thermal contraction. These aforementioned conditions influence the specimen thermal performance, which includes HDT, glass-transition temperature (*Tg*), and high-temperature stability, and dimensional response, which includes shrinkage, warping, and volumetric changes. Dimensional distortion produced by thermal treatments can be efficiently limited through constrained annealing, but this type of treatment can limit stress relaxation. Therefore, reaching an adequate equilibrium between dimensional stability and thermomechanical optimization is extremely important. Similarly, an excessive temperature setpoint or dwell time may promote the degradation, embrittlement, or discoloration of the polymer matrix, which highlights the importance of defining an adequate processing window and using gradual heating, geometric fixtures, and application-specific thermal conditions.

### 7.1. Glass Transition Temperature and Heat Deflection Temperature

Reported changes in *Tg* after annealing are generally modest in absolute terms but mechanistically informative: for a graphene-reinforced PLA composite, annealing at 120 °C increased crystallinity from approximately 4.8% to 59%, while *Tg* increased only slightly, from 57.6 °C to 58.2 °C, consistent with the physical picture that crystalline regions restrict amorphous-phase chain mobility (raising *Tg* marginally) without themselves melting or altering the fundamentally amorphous-phase nature of the glass transition [86]. More practically significant for service temperature range is HDT, which is reported to increase substantially after annealing in several thermoplastic composite systems, and which is explicitly cited as a primary motivation for annealing in application-oriented guidance on heat-resistant AM materials [69]. Fiber reinforcement itself interacts with this effect: carbon fiber has been reported to act as a nucleating agent that both increases HDT and widens the usable annealing temperature window in PLA-CF relative to neat PLA, because fiber-constrained polymer chain mobility allows the matrix to tolerate higher treatment temperatures before excessive distortion occurs [42]. This same interaction was reflected in Section 6.1, where annealed PAHT-CF retained a tensile-strength advantage over untreated material even when tested up to 150 °C, indicating that annealing-induced improvements are not confined to room-temperature performance but extend into the elevated-temperature service range relevant to under-hood, tooling, and other thermally demanding applications [72].

### 7.2. Thermal Degradation and Stability

Thermogravimetric analysis (TGA) of heat-treated AM-PMCs generally shows that annealing at temperatures below the matrix degradation onset does not measurably reduce thermal stability, and in fiber-reinforced systems, the reinforcement itself improves high-temperature mass retention: polyamide-6/glass-fiber composites retained roughly 26% of their mass at 350 °C in TGA testing, compared to only 3% and 5% retention for neat PA-6 and PA-6/graphene-oxide composites respectively, indicating that glass fiber reinforcement provides a thermal stability benefit largely independent of, but complementary to, any heat-treatment-induced changes in the matrix [87,88]. Annealing protocols in the literature are, as a rule, deliberately bounded well below degradation onset temperatures identified by TGA, so reported degradation-related risks in Section 4 and Section 5 (e.g., discoloration or embrittlement in post-cured photopolymers, Section 4.4) are more commonly associated with prolonged or excessive-temperature treatment outside recommended windows than with properly bounded annealing protocols.

### 7.3. Dimensional Stability: Shrinkage, Warping, and Volumetric Change

Dimensional response to heat treatment is the clearest manifestation of the stress-relaxation/crystallization coupling effect discussed in Section 5.5, and is reported with considerable variability depending on different conditions, such as material, treatment method, and geometric constraint. For instance, in composites reinforced with continuous carbon fiber, increasing the temperature setpoint fixed during 4 h progressively produced thermal shrinkage in both width and thickness for the neat matrix and the fiber-reinforced composite. Moreover, the fiber-reinforced composite exhibited a generally reduced shrinkage response when compared to the unreinforced matrix due to fiber constraint on matrix contraction [29].

Different studies report that the medium used for heat transfer significantly affects the specimen’s dimensional outcome, as sand-bath annealing produced improved dimensional stability than conventional oven annealing across most carbon-fiber composites, with oven-annealed ABS-CF showing deviations along the build (*Z*) axis of approximately 18% at the highest annealing temperature tested (125 °C), compared to a deviation of around 7.6% for the direction, material, and temperature under sand-bath annealing. This significant modification was attributed to the more gradual and uniform heat transfer provided by a granular medium compared to convection oven heating [4]. At the end, warpage in unreinforced 3D-printed polyamide parts has been reported as high as 72.6%, a magnitude that can be reduced to as little as 3.4% through gradient/multi-material structural design that offsets interlayer shrinkage stress, illustrating that heat-treatment-related dimensional response cannot be considered independent of the part’s structural and material design, only its post-processing protocol [2].

Similar effects have been observed in DIW carbon-fiber/thermoset composites. Saito et al. [89] reported appreciable dimensional differences between the as-printed green body and the final cured state, particularly in specimen thickness, while Fernandez et al. [90] suggested that curing-induced volumetric changes and the associated residual internal stresses may contribute to discrepancies in structural response. Constrained or fixture-based annealing (Section 4.2) is the direct post-processing-side countermeasure to this variability, trading some of the stress-relief benefit for tighter dimensional control, and dedicated dimensional-precision studies of thermally post-processed PLA report that carefully tuned annealing windows can simultaneously increase dimensional precision and confer compatibility with subsequent high-temperature processing such as autoclave sterilization, without the warpage penalty associated with uncontrolled annealing.

### 7.4. Surface Quality: Roughness, Stair-Stepping, and Topographical Evolution

Heat treatments can also modify the surface topography of AM-PMCs, although this aspect has received considerably less attention than bulk mechanical and dimensional properties. The characteristic stair-stepping effect and layer-induced surface roughness originate primarily from the layer thickness, deposition path, bead geometry, and incomplete fusion between adjacent roads [91,92]. Through increasing polymer-chain mobility, reducing residual stresses induced by material deposition, and promoting local viscous flow and interlayer consolidation, thermal treatment may be able to modify the overall surface quality of AM-PMC composites [93]. Thermal post-processing under controlled conditions can reduce localized surface irregularities and improve surface continuity; however, excessive annealing temperature or dwell time can generate non-uniform shrinkage, warping, bead deformation, or matrix redistribution, which might potentially increase surface waviness and dimensional deviations. The addition of fiber reinforcement in additive specimens fabricated by means of AM adds further complexity to the overall material response, since the reinforcement restricts matrix flow and may locally increase surface roughness or even promote fiber exposure [94]. Therefore, surface roughness should be evaluated in conjunction with dimensional accuracy and stair-step geometry, rather than as an isolated parameter, particularly when tight tolerances, aerodynamic surfaces, sealing interfaces, or subsequent machining are required for the intended application of heat-treated AM-PMCs. This aspect remains comparatively underexplored in the literature, highlighting the need for standardized characterization of roughness parameters (e.g., Ra, Rq, Rz), waviness, and layer-profile evolution before and after thermal treatment.

### 7.5. Quantitative Comparison of Coupled Crystallinity, HDT, and Dimensional Response

The individual studies discussed in Section 6 and Section 7 report heat-treatment outcomes across different property categories using different temperature–time protocols, which makes the underlying trade-off between crystallinity, HDT, dimensional stability, and mechanical response difficult to appreciate from a section-by-section reading. Table 3 consolidates the reported values from the primary studies cited above into a single comparative frame, listing for each study the treatment temperature and time, the reported crystallinity change, the HDT or *Tg* change, the reported dimensional response (shrinkage, warpage, or *Z*-axis deviation), and the associated mechanical gain, using the same references already discussed in Section 6.1, Section 6.2, Section 6.3 and Section 6.4 and Section 7.1, Section 7.2 and Section 7.3.

Several patterns emerge from the tabulated data. First, the very large crystallinity gains reported for annealing in the vicinity of *Tg* (for example, PLA/graphene at 120 °C, where crystallinity is reported to rise from approximately 4.8% to 59%) are accompanied by only marginal shifts in *Tg* (0.6 °C in the same study), consistent with the Section 7.1 interpretation that the glass transition is fundamentally an amorphous-phase phenomenon and is therefore only weakly displaced by densification of the crystalline fraction; the practical service-temperature benefit of annealing arises through HDT rather than *Tg*. Second, the largest mechanical gains—ILSS improvements of the order of 100–500% and, in the pressure-assisted PEEK case, up to approximately six-fold—occur at treatment temperatures near or above *Tm*, and the same protocols are those most likely to produce measurable *Z*-axis expansion when unconstrained (Section 7.3). Third, the studies that explicitly optimize for balanced mechanical performance report intermediate treatment temperatures (approximately 90 °C, 120 min for neat PLA in [76]) at which crystallinity gains are moderate and dimensional distortion is not reported as a limiting factor. Finally, and most importantly for future work, Table 3 shows that a majority of the reviewed studies report a mechanical outcome but do not report the coupled crystallinity, HDT, and dimensional data required to place their result on a common axis, which is a specific instantiation of the standardization gap discussed in Section 9.1; closing this gap by requiring simultaneous reporting of thermal, dimensional, and mechanical outcomes for a given treatment protocol would allow the tabulated data to be extended into a genuine process–property design chart.

### 7.6. Synthesis

Thermal and dimensional outcomes of heat treatment are best understood as two faces of the same underlying stress-relaxation and crystallization mechanisms discussed in Section 5: processes that improve HDT and elevated-temperature mechanical retention (Section 7.1) generally do so through the same crystallization and interlayer-consolidation pathways that also drive shrinkage and, in unconstrained geometries, warping (Section 7.3). This creates a practical design trade-off for any heat treatment protocol targeting improved thermal performance: maximizing HDT and crystallinity gain favors higher treatment temperatures and longer dwell times, while minimizing dimensional distortion favors either lower temperatures/shorter times or the use of constraining fixtures, gradual heating media, or structurally compensating design features, a trade-off that recurs across virtually all thermoplastic AM-PMC systems reviewed here (Figure 6) and that motivates the process-parameter optimization studies discussed further in Section 9.

## 8. Comparative Analysis Across Reinforcement Types and Matrix Systems

Section 5, Section 6 and Section 7 analyzed the different properties that can be modified by means of heat treatment acts. Thus, this section is dedicated to synthesizing those findings across the two dimensions that exert the most important influence on thermal treatment response, which are matrix chemistry (amorphous vs. semicrystalline thermoplastic, thermoset/photopolymer) and reinforcement type (short fiber, continuous fiber, particulate, natural fiber), to identify where the literature shows consistent trends as opposed to where material-specific behavior dominates.

### 8.1. Matrix-Driven Differences

Semicrystalline thermoplastics (PLA, PA, PP, PEEK, PEKK, PPS) show the most consistent and mechanistically well-characterized response to annealing, because the same treatment window simultaneously drives crystallization, stress relaxation, and interlayer healing (Section 5) [5]. Amorphous thermoplastics (ABS, standard PC) rely on stress relaxation and interlayer diffusion alone, without a crystallization contribution, and consequently show comparatively smaller HDT gains from heat treatment relative to semicrystalline matrices, though interlayer-bonding-driven strength gains are still reported [95]. On the other hand, thermoset and photopolymer systems (VPP resins, epoxy resin-based systems) respond to heat (and/or UV) treatment through a completely different mechanism. In this case, heat treatment promotes the completion of cure/crosslinking reactions rather than crystallization, and is consequently treated with post-cure protocols (Section 4.4) rather than annealing; thermoset and photopolymer response to heat treatment is restricted by the resin’s cure kinetics and reinforcement-dependent light or heat penetration rather than by *Tg*-referenced thermal mobility. Quantitative envelopes for the crystallinity–HDT–dimensional–mechanical trade-off across the matrix families discussed in this subsection are consolidated in Table 3 (Section 7.5).

### 8.2. Reinforcement-Driven Differences

Reinforcement type modulates the heat-treatment response along at least three distinguishable axes reported across the studies surveyed in Section 6 and Section 7:

- **Short-fiber vs. continuous-fiber composites.** Short-fiber composites show fiber-orientation-dependent, generally moderate gains from annealing (tensile, flexural, impact improvements broadly in the 10–60% range, Section 6.1 and Section 6.2), while continuous-fiber composites, particularly when combined with pressure-assisted treatment, show large ILSS enhancements (from 100% to >500%, as described in Section 6.3). This phenomenon is attributed to a more geometrically consistent interlayer weld zone towed by continuous fiber, where pressure-assisted consolidation can efficiently act.
- **Carbon vs. glass fiber.** Carbon-fiber-reinforced composites generally display a higher baseline stiffness and a stronger and more consistent ILSS response to thermal treatment (peaking near 196–203% of baseline near 170 °C, Section 6.3). On the other hand, glass-fiber-reinforced composites generally exhibit a weaker and less consistent response, which can even lead to an ILSS degradation at the highest exposure temperature tested, usually attributed to thermal microcracking rather than beneficial reconsolidation [74]. This asymmetry is consistent with carbon fiber’s established higher stiffness-to-weight ratio and closer thermal expansion match to typical engineering matrices relative to glass fiber [74], while glass fiber is separately reported to offer greater impact toughness and lower cost in the as-printed state, a trade-off that is not eliminated by heat treatment [96].
- **Fiber presence itself vs. unreinforced matrix.** Across nearly all systems reviewed, fiber-reinforced composites show a distinct—and not uniformly favorable—starting point relative to the unreinforced matrix: short-carbon-fiber reinforcement has been reported to reduce interlayer tensile strength relative to neat matrix in the as-printed state (attributed to fiber-increased melt viscosity slowing interlayer diffusion), a deficit that annealing was shown to substantially recover or eliminate [87]. This indicates that heat treatment is, in a meaningful sense, more mechanistically necessary for fiber-reinforced AM-PMCs than for unreinforced polymer prints, since fiber reinforcement both improves ultimate performance ceiling and degrades as-printed interlayer quality.
- **Natural and particulate reinforcement.** Comparatively few heat-treatment studies address natural-fiber (e.g., bamboo, sugar palm, wood flour) or particulate/nanofiller (graphene, carbon nanotubes, boron nitride) systems relative to synthetic continuous or short-fiber composites [97]; where reported, natural-fiber and nanofiller systems show property and thermal-stability responses that are more strongly governed by filler-matrix chemical interaction, which can be highly affected by chemical surface treatments, such as alkali or silane treatments, rather than by the heat-treatment protocol itself. This suggests a comparatively underexplored area when compared to carbon- and glass-fiber systems (identified further as a gap in Section 9).

Table 4 consolidates the reinforcement- and matrix-level trends discussed above, drawing on the quantitative findings reported in Section 5, Section 6 and Section 7.

This comparative structure indicates that no single heat-treatment protocol generalizes across material systems: the treatment temperature, dwell time, and whether pressure assistance is warranted must be selected according to the dominant as-printed deficiency of the specific matrix-reinforcement combination: porosity- and interlayer-limited for short-fiber thermoplastics, crystallinity-limited for high-performance semicrystalline systems, and cure-limited for thermosets, a conclusion that directly motivates the discussion of standardization gaps in Section 9.

## 9. Current Challenges and Research Gaps

**Figure 7:**
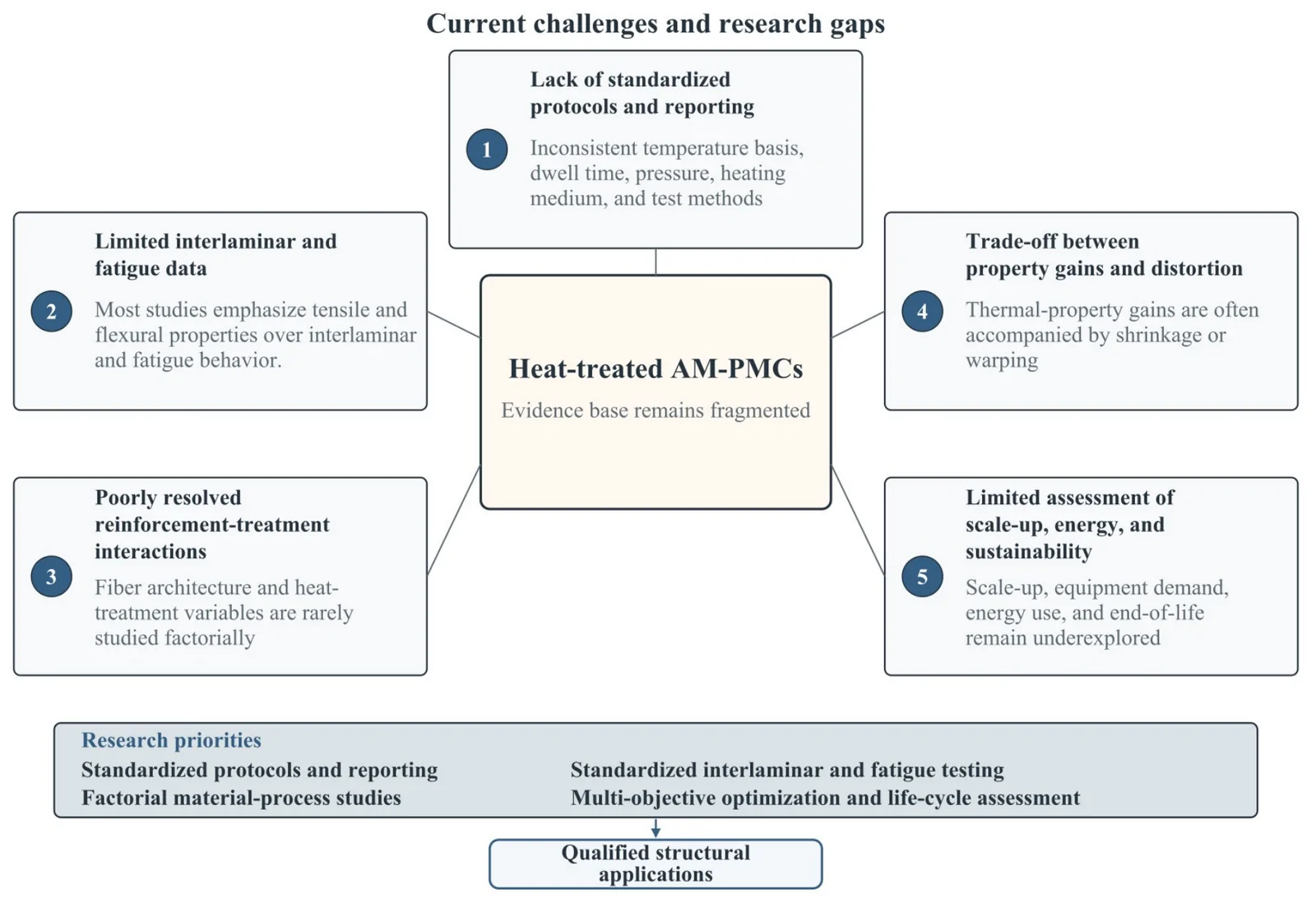
Schematic description of challenges and research priorities associated with heat treatment of additively manufactured polymer matrix composites (AM-PMCs).

Synthesizing across Section 3, Section 4, Section 5, Section 6, Section 7 and Section 8, five recurring limitations constrain the field’s ability to translate heat-treated AM-PMCs into qualified structural applications. Figure 7 links the principal characteristics of the as-printed material with free, constrained, and pressure-assisted heat-treatment strategies, highlighting the associated microstructural evolution and reported changes in mechanical, thermal, fatigue, and dimensional performance. The lower section summarizes five major research gaps: standardization, limited interlaminar and fatigue characterization, reinforcement-dependent optimization, property and dimensional stability trade-offs, and scalability/energy/sustainability, and identifies corresponding research priorities required to establish reproducible, scalable, and application-oriented heat-treatment protocols for structural AM-PMCs.

### 9.1. Lack of Standardized Treatment Protocols and Testing Metrics

The single most consistent limitation identified across this review is the absence of standardized heat-treatment protocols and reporting conventions. Treatment temperature is variably referenced to *Tg*, *Tm*, or a manufacturer-recommended value; dwell time ranges from minutes to many hours across studies addressing ostensibly similar material systems; pressure magnitude, geometric constraints, and heating medium (oven, sand bath, autoclave, silicone-mold HIP) are rarely held constant even within single-variable studies. This is compounded by an acknowledged lack of accepted standards and consistent testing methods for evaluating AM post-processing outcomes more broadly, which limits direct cross-study comparison and complicates the extraction of general design rules from the literature [121]. A specific consequence, evident in Section 6, is that the same nominal treatment (“annealing”) can be reported as improving or degrading a given property, depending on unreported or under-specified variables such as atmosphere, moisture conditioning, or geometric constraints (Section 5.3 and Section 5.4).

### 9.2. Underrepresentation of Interlaminar and Fatigue Characterization Relative to Quasi-Static Bulk Testing

While Section 6.3 and Section 6.4 identified interlaminar shear and fatigue as the property categories most sensitive to heat treatment, they remain comparatively underrepresented relative to tensile and flexural characterization in the broader literature. Given that interlayer bonding is the dominant failure-limiting feature of AM parts in general, this imbalance leaves an incomplete picture of treatment efficacy for the loading modes most likely to govern real-component failure.

### 9.3. Limited Coupling Between Reinforcement Type and Treatment Optimization

Section 8 showed that reinforcement type (short vs. continuous fiber, carbon vs. glass, synthetic vs. natural) substantially modulates the heat-treatment response, yet few studies systematically vary both reinforcement type and treatment parameters within a single, controlled experimental design. Most published work instead treats a single material system in isolation, which limits the field’s ability to derive reinforcement-general design principles and instead produces a fragmented, material-specific body of empirical findings.

### 9.4. Trade-Off Management Between Property Improvement and Dimensional/Thermal Side Effects

Section 7.4 identified an inherent compromise between maximizing crystallinity/HDT gains and minimizing shrinkage/warping. Relatively few studies explicitly optimize across this trade-off (e.g., via multi-objective statistical design), and fixture-based or constrained-annealing solutions (Section 4.2), while conceptually straightforward, are inconsistently reported with the quantitative dimensional-control data needed to generalize their effectiveness across geometries.

### 9.5. Scalability, Energy Cost, and Sustainability Considerations

Beyond the materials-science limitations above, broader adoption of heat-treated AM-PMCs is also constrained by process-economic and sustainability factors: systematic reviews of polymer-based smart and composite materials in AM identify persistent gaps in scalability, regulatory standardization, and end-of-life/recyclability considerations that extend to post-processing steps such as heat treatment, alongside concerns about the added energy consumption and production time that thermal or pressure-assisted post-processing introduces relative to as-printed parts [122,123]. These considerations are particularly important in pressure-assisted methods (HIP, hot pressing; Section 4.3), which can yield greater improvements in AM-PMCs (Section 6.3), though this greater improvement can be achieved with more sophisticated equipment complexity and higher energy demands than free-oven annealing.

A further sustainability consideration, which is largely absent from the current AM-PMC heat-treatment literature, is the effect of the imposed thermal history on the recyclability of the treated part at end-of-life. For thermoplastic-matrix composites, every additional thermal excursion above *Tg*, including annealing and pressure-assisted consolidation, contributes to cumulative thermo-oxidative aging and, in the case of hydrolytically sensitive matrices such as PLA and PA, to hydrolytic chain scission if moisture is not adequately controlled during treatment. The resulting reduction in matrix molecular weight lowers the number of mechanical-recycling cycles that reground feedstock can subsequently tolerate before its melt viscosity and impact properties fall below the acceptance window for reprocessing. The same annealing protocols that increase crystallinity for service-temperature benefit (Section 7.1) also shift the crystallization kinetics and melt behavior of any reground material relative to virgin feedstock, complicating its use as a drop-in extrusion or FFF feedstock and typically requiring blending with virgin polymer to restore processability. For thermoset and photopolymer systems, post-curing (Section 4.4) drives the network toward its maximum crosslink density, which by definition precludes melt-based recycling and channels these materials toward mechanical grinding as filler, chemical solvolysis, or pyrolysis for fiber recovery—routes that are less mature and, at present, less economically attractive than melt reprocessing of thermoplastics.

Finally, pressure-assisted consolidation (Section 4.3) tightens the fiber–matrix interface in continuous-fiber composites and is therefore beneficial for as-manufactured mechanical performance, but the same tighter consolidation complicates fiber-matrix separation during end-of-life fiber recovery, a consideration that is particularly relevant for carbon-fiber systems, where reclaimed fibers are the highest-value recycling output. Systematic life-cycle-assessment-style comparisons of the “as-printed → treated → in-service → recycled” chain for AM-PMCs are, to the best of the authors’ knowledge, not yet available in the reviewed literature, and represent a natural extension of the sustainability-integrated process-selection direction outlined in Section 10.

## 10. Future Perspectives

Based on the research gaps identified in Section 9, several directions appear to be well-positioned to produce an important development in the field:

- **Standardized, multi-variable protocol reporting.** Adoption of a common minimum reporting standard for heat-treatment studies, treatment temperature normalized to *Tg*/*Tm*, dwell time, heating/cooling rate, atmosphere, moisture-conditioning state, and constraint condition, would substantially improve cross-study comparability and enable meta-analysis-style synthesis across the fragmented literature identified in Section 9.1.
- **Coupled reinforcement–treatment optimization studies.** Statistically designed experiments (e.g., Design of Experiments, Taguchi, response-surface methodology) that simultaneously vary reinforcement type/content and treatment parameters within a single study, of the kind already demonstrated for single-material systems (Section 6.2), could be extended across reinforcement families to directly test the comparative trends synthesized in Section 8.
- **Interlaminar- and fatigue- focused characterization.** Given the outsized sensitivity of interlaminar and fatigue properties to heat treatment (Section 6.3 and Section 6.4), expanding standardized short-beam shear, mode I/II fracture toughness, and cyclic-loading characterization specifically for heat-treated AM-PMCs would better represent the loading conditions most relevant to structural qualification.
- **In situ and hybrid consolidation strategies.** Emerging approaches that integrate consolidation directly into the print process—such as in-nozzle infrared preheating combined with roller compaction (Section 4.3)—point toward a broader trend of shifting some heat-treatment benefit upstream into the build process itself, potentially reducing the need for lengthy, energy-intensive post-print treatment while retaining its interlayer-healing benefit.
- **Machine-learning-assisted process-property modeling.** Given the high dimensionality of the treatment parameter space (temperature, time, pressure, atmosphere, constraint, material system) and the coupled, sometimes opposing mechanisms identified in Section 5.5, data-driven modeling approaches trained across a standardized reporting framework (per the first recommendation above) could help predict optimal treatment windows for new material combinations without requiring exhaustive experimental screening for each new system.
- **Sustainability-integrated process selection.** Future work should evaluate heat-treatment protocols not only by mechanical/thermal performance gain but also by energy cost and compatibility with recyclability and end-of-life goals, aligning post-processing decisions with the broader sustainability gaps identified for polymer-based AM materials [123].

## 11. Conclusions

This review examined the effect of post-fabrication heat treatment on additively manufactured polymer matrix composites, synthesizing findings across process types, treatment methods, underlying microstructural mechanisms, and resulting mechanical, thermal, and dimensional performance. Several conclusions emerge from this synthesis:

- The as-printed microstructure of AM-PMCs, regardless of whether produced by material extrusion, vat photopolymerization, powder bed fusion, or material jetting, is consistently characterized by a metastable or non-equilibrium state relative to conventionally processed composites: sub-equilibrium crystallinity or incomplete cure, interlayer porosity, and locked-in residual stress (Section 3). Heat treatment is the primary post-processing route by which this state is deliberately modified, and the specific treatment strategy that is appropriate depends on which deficiency dominates for a given material-process combination—porosity- and interlayer-limited for short-fiber thermoplastics, crystallinity-limited for high-performance semicrystalline systems, and cure-limited for thermosets and photopolymers (Section 4 and Section 8).
- Four coupled physicochemical mechanisms, crystallization, interlayer/interface healing, porosity evolution, and residual stress relaxation, govern the property changes observed after heat treatment (Section 5), and their relative dominance, rather than treatment temperature or time alone, explains much of the variability reported across the literature. Properties governed strongly by interlayer integrity, particularly interlaminar shear and joint-level performance, exhibit the largest and most consistently reported improvements after heat treatment. Fatigue performance also appears sensitive to interlayer healing and porosity reduction; however, the limited number and heterogeneity of available studies prevent establishing a generalized quantitative improvement in fatigue life. Several studies have reported cases with an enhancement exceeding 100% for conventional oven annealing, and improvements that can reach an order of magnitude for pressure-assisted treatments, while bulk tensile and flexural properties show smaller and more treatment-condition-dependent improvements (Section 6).
- Heat treatment effects are not solely related to mechanical performance, as the same crystallization and stress-relaxation mechanisms that increase heat deflection temperature and elevated-temperature mechanical retention are the same ones that drive shrinkage and, in unconstrained geometries, warping (Section 7). This coupling constitutes a practical design trade-off that any heat-treatment protocol must navigate, and it is addressed unevenly across the literature, through constrained annealing, gradual heating media, or structurally compensating design—without a generalized, quantitatively validated solution.
- Reinforcement type and matrix chemistry substantially modulate the heat-treatment response (Section 8): continuous-fiber composites benefit disproportionately from pressure-assisted consolidation; carbon fiber composites show a stronger and more consistent interlaminar response to thermal exposure than glass fiber composites, and fiber reinforcement itself, while raising the achievable performance ceiling, degrades as-printed interlayer quality relative to the unreinforced matrix, making heat treatment arguably more mechanistically necessary for reinforced systems than for neat polymer prints.
- Generalizing these findings into transferable design rules for the field remains difficult, given the lack of standardized treatment protocols and reporting conventions. In addition, the overreliance on quasi-static bulk characterization, in comparison to interlaminar and fatigue testing, limited studies that combinedly vary reinforcement type and treatment parameters. Also, our efforts have identified an underdeveloped consideration of the energy consumption, scalability, and sustainability costs of thermal and pressure-assisted post-processing (Section 9). Addressing these gaps through standardized multi-variable reporting, coupled reinforcement-treatment optimization studies, expanded interlaminar/fatigue characterization, in situ or hybrid consolidation strategies, and data-driven process-property modeling (Section 10) represents the most direct path toward qualifying heat-treated AM-PMCs for the structural and semi-structural applications that motivate continued interest in this technology.

## Figures and Tables

**Table 1:** Dominant as-printed defects in PMCs obtained through the AM processes.

AM Process	Dominant As-Printed Defects	Main Origin	Schematic Structure
FFF/FDM	Interlayer/raster porosity, incomplete interlayer bonding, anisotropy, residual stress	Bead deposition, limited chain diffusion, rapid cooling	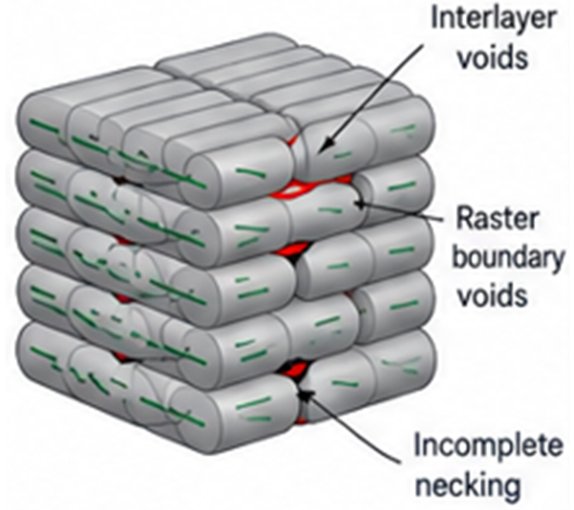
VPP	Incomplete cure, reinforcement-induced voids, cure heterogeneity, shrinkage	Light attenuation/scattering and incomplete polymerization	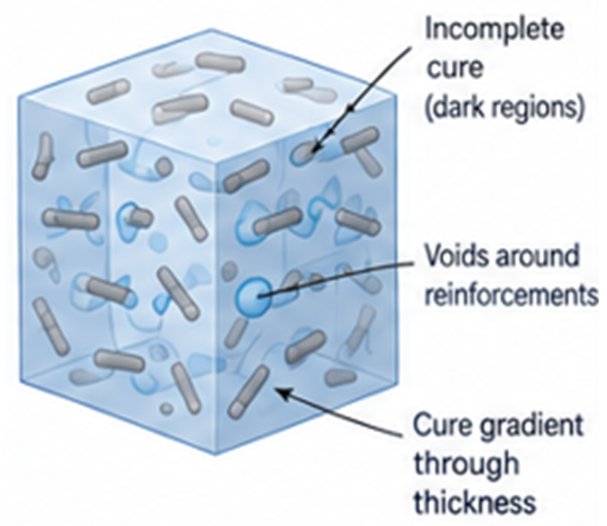
SLS	Inter-particle porosity, incomplete fusion, filler-induced heterogeneity	Incomplete powder sintering and disrupted melt flow	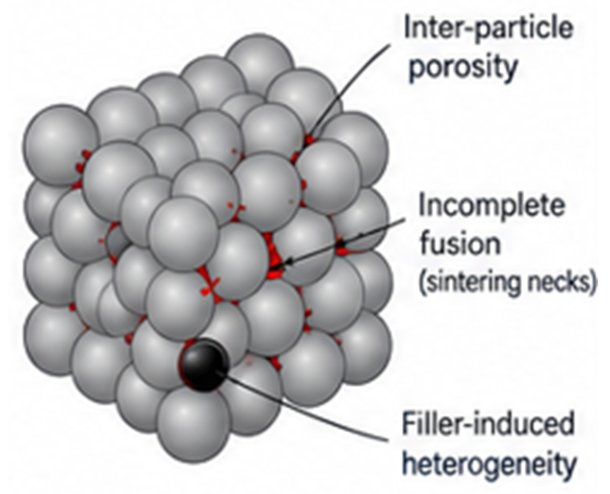
MJ	Incomplete polymerization, voids/porosity between droplets, weak interlayer bonding, dimensional inaccuracy/shrinkage	Droplet solidification, limited interlayer diffusion, UV light scattering/absorption, and process parameters such as droplet spacing and curing dose	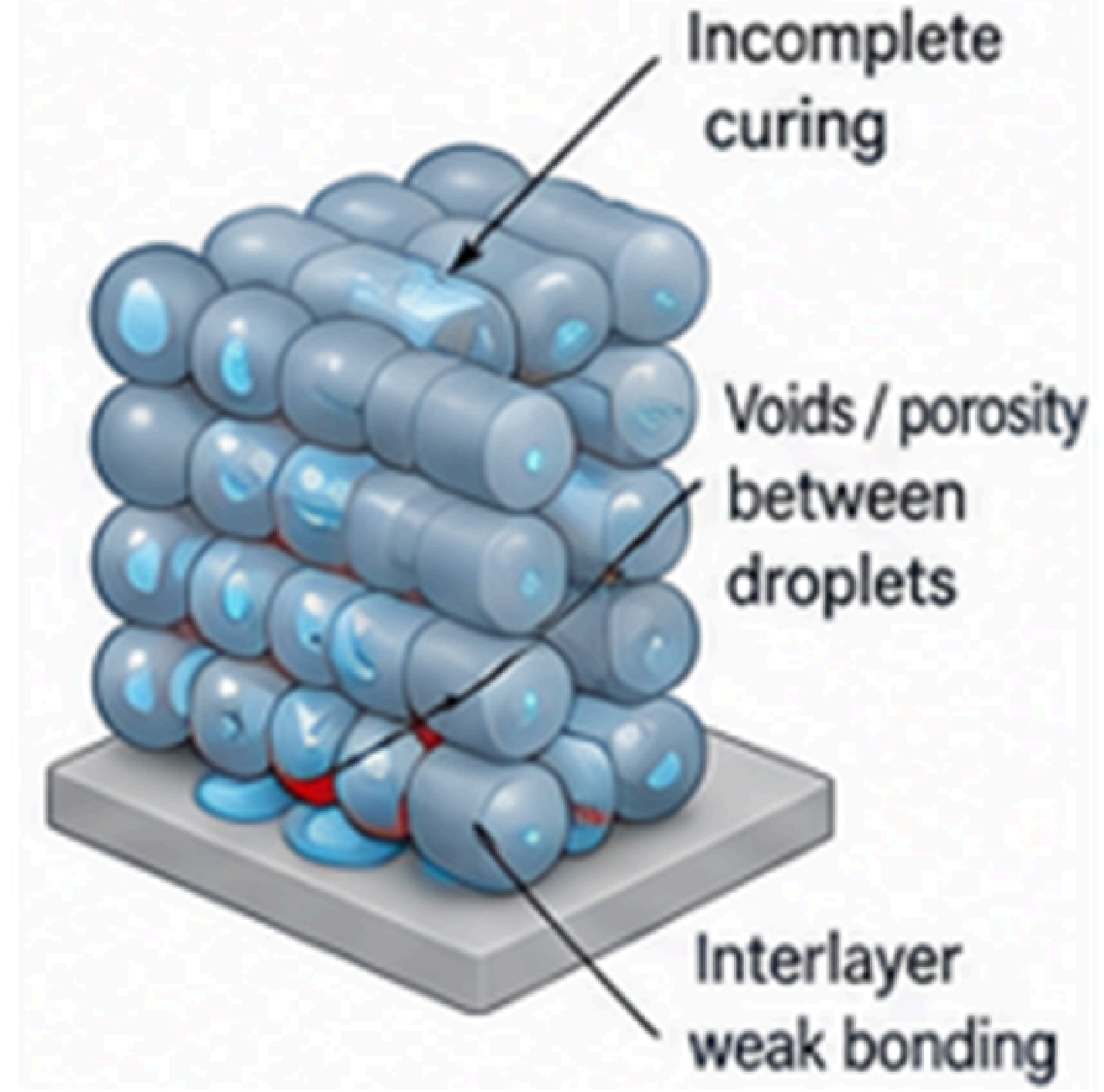

**Table 2:** Heat-treatment methods for AM-PMCs, organized into four mechanistically distinct categories.

Category	Sub-Variant	Primary Material Class	Dominant Mechanism	Principal Trade-Off	Ref.
Post-print thermal treatment	Free (unconstrained) annealing	Semicrystalline thermoplastics; short-fiber composites	Stress relaxation, recrystallization, limited interbead healing (heat only)	Warpage/dimensional change; possible secondary porosity	[40,49,50,51,52,53,54,55]
	Constrained annealing	Semicrystalline thermoplastics (dimensionally sensitive parts)	Same as free annealing under geometric restraint	Reduced stress relief relative to free anneal	[56,57,58,59]
Thermo-mechanical consolidation	Hot isostatic pressing (HIP); silicone-mold HIP; autoclave	Continuous- and short-fiber thermoplastic composites	Pressure-assisted void closure + interlayer autohesion (heat + isostatic pressure)	Equipment complexity; geometry constraints; possible fiber-bed disruption	[45,46,60,61,62]
	Uniaxial hot pressing/platen pressing	Continuous-fiber laminates; flat/near-net-shape parts	Directional consolidation and densification (heat + uniaxial pressure)	Restricted to simple/flat geometries	[47]
Post-curing	UV, thermal, or combined UV + thermal post-cure; electron-beam post-cure	Photopolymer and thermoset-based systems (VPP, DIW, MJ)	Completion of monomer conversion/crosslinking (photochemical or thermal, not melt-based)	Discoloration, embrittlement, or biocompatibility shifts at high dose/temperature	[30,39,48,63]
In-situ thermal (and thermo-mechanical) processing	In-nozzle IR preheating; roller compaction integrated at the print head; laser-assisted deposition	Continuous- and short-fiber thermoplastic composites (FFF/FDM)	Local reheating and/or compaction of the freshly deposited road during build (mechanism activated concurrently with deposition rather than after solidification)	Requires modified hardware; effect distributed across the build; less mature than post-print routes	[49]

**Table 3:** Quantitative comparison of reported heat-treatment outcomes on crystallinity, HDT/*Tg*, dimensional response, and mechanical performance across representative AM-PMC systems. “Not reported” indicates that a specific quantity was not reported in the cited source.

Material System	Treatment (T, t, Medium)	Δ Crystallinity	Δ HDT/*Tg*	Dimensional Response	Mechanical Gain	Ref.
CF-PETG (FFF)	Manufacturer-recommended anneal	Increase reported (magnitude n/r)	Not reported	Not reported	Hardness +≈20%; tensile +8–25%; impact +5–25%	[41,65]
PAHT-CF (FFF)	80 °C, 12 h, oven, free	Not quantified	HDT increase inferred (strength retained at 150 °C)	Not reported	Tensile +50% (RT); +17.5% at 150 °C; strain at failure −70%	[73]
ABS-CF (FFF)	125 °C, oven vs. sand-bath	Not reported	Not reported	*Z*-axis deviation: 18% (oven) vs. 7.6% (sand-bath)	Comparable strength gains; medium controls distortion	[42]
PA6/GF (FFF)	Free anneal, sub-degradation	Not quantified	TGA mass at 350 °C: 26% vs. 3% (neat PA6)	Not reported	High-T stability contributed largely by GF	[29]
Short + continuous CF/PEEK (FFF)	Optimized anneal (near *Tm*)	Increase reported (magnitude n/r)	HDT increase reported	Not reported	ILSS 5.78 → 39.05 MPa (≈+575%)	[65]
CCF/aramid/GF Onyx (FFF)	170 °C peak; up to 200 °C, free	Not directly quantified	Not reported	Not reported	CFRP ILSS +196%; AFRP +203%; GFRP −16% at 200 °C	[77]
CCF composite (FFF)	180 °C, free	Not quantified	Not reported	Not reported	Max load +83.6% (5% vf); +86.8% (15% vf)	[78]
CCF/PEEK (in-situ IR + rollers)	In-process, optimized	Not quantified	Not reported	Not reported	ILSS +117%	[79]
Acrylate- resin + TiO_2_ (SLA)	400–1000 °C, 1 h	Increase reported (magnitude n/r)	*Tg*: 71.72 → 79.62 °C	Not reported	Tensile +103% Modulus +32%	[81]
Acrylate- resin + CNT (DLP)	UV post-curing, 30 min, oven	Not reported	*Tg*: 40 → 52 °C	Not reported	Tensile +40% Modulus +32%	[82]
PA + GF (SLS/MJ)	124–173 °C, 5 h	Increase 24.54% → 29.07%	*Tm*: 179 → 184 °C	Not reported	Tensile +22.8% Modulus +30.6%	[83]
CCF thermoplastic (platen-pressed)	Hot pressing after print	Not quantified	Not reported	Not reported	Fatigue life > 10^6^ cycles at 80% UTS (vs. ≈5 × 10^4^ at 90%)	[84]
Graphene/PLA (FFF)	120 °C, oven, free	≈4.8% → 59% (Χc)	*Tg*: 57.6 → 58.2 °C (+0.6 °C)	Not reported quantitatively; free anneal	Stiffness increase reported	[85]

**Table 4:** Reinforcement types and matrix systems in additive manufacturing.

Material System	Dominant Heat-Treatment Mechanism	Typical Property Gain Reported	Principal Limitation/Risk	Ref.
Unreinforced semicrystalline thermoplastic (PLA, PA)	Crystallization + stress relaxation + interlayer healing	Tensile +20–60%; flexural up to +58%; HDT increase	Warping/shrinkage if unconstrained; diminishing or reversing gains with excessive dwell time	[41,69,72,73,98,99,100,101]
Short-fiber composite (CF/GF-PLA, -PETG, -PA, -ABS)	Interlayer healing dominant; matrix crystallization secondary	Tensile +8–200%+ (highly variable); interlayer tensile strength restored toward neat-matrix levels	As-printed interlayer deficit larger than unreinforced matrix; fiber increases melt viscosity, slowing healing kinetics	[78,85,102,103,104,105]
Continuous-fiber composite (CCF/PEEK, CF/Onyx, CCFRC)	Interlayer healing + pressure-assisted void closure	ILSS +100% to >500%; maximum load +80–90%	Requires pressure-assisted (HIP/hot-press) treatment for largest gains; glass fiber variant shows weaker/inconsistent response	[64,75,76,106,107,108]
High-performance semicrystalline (PEEK, PEKK, PPS) composites	Crystallization (large as-printed deficit) + interlayer healing	ILSS increase up to ~6×; substantial HDT/elevated-temp retention	Narrow, high-temperature processing window; higher equipment/energy cost	[64,109,110,111,112,113,114,115]
Thermoset/photopolymer (VPP resin, epoxy) composites	Completion of cure/crosslinking (post-cure)	Tensile/strength increase tied to conversion increase	Discoloration, embrittlement, or biocompatibility shift at high UV dose or temperature	[30,39,48]
Natural-fiber/particulate-filled composites	Filler-matrix interaction-dependent; crystallization/stress relaxation secondary	Reported gains material- and treatment-specific; limited standardized data	Comparatively underexplored; property response entangled with fiber surface treatment	[116,117,118,119,120]

## Data Availability

The original contributions presented in this study are included in the article. Further inquiries can be directed to the corresponding authors.

## Abbreviations

The following abbreviations are used in this manuscript:

AM	Additive Manufacturing
PMC	Polymer Matrix Composite
FFF	Fused Filament Fabrication
FDM	Fused Deposition Modeling
VPP	Vat Photopolymerization
SLA	Stereolithography
DLP	Digital Light Processing
SLS	Selective Laser Sintering
DIW	Direct Ink Writing
MJ	Material Jetting
PEEK	Polyether Ether Ketone
PEKK	Polyether Ketone Ketone
PPS	Polyphenylene Sulfide
*Tg*	Glass Transition Temperature
*Tm*	Melting Temperature
HDT	Heat Deflection Temperature
HIP	Hot Isostatic Pressing
ILSS	Interlaminar Shear Strength
PLA	Polylactic Acid
PA	Polyamide (nylon)
PP	Polypropylene
CF	Carbon Fiber
GF	Glass Fiber
Ra	Arithmetic Average Roughness
Rq	Root Mean Square Roughness
Rz	Average Maximum Height
DSC	Differential Scanning Calorimetry
XRD	X-ray Diffraction
SEM	Scanning Electron Microscopy
µCT	Micro-Computed Tomography
